# Plastomes of limestone karst gesneriad genera *Petrocodon* and *Primulina*, and the comparative plastid phylogenomics of Gesneriaceae

**DOI:** 10.1038/s41598-022-19812-2

**Published:** 2022-09-22

**Authors:** Chia-Lun Hsieh, Wei-Bin Xu, Kuo-Fang Chung

**Affiliations:** 1 Research Museum and Herbarium (HAST), Biodiversity Research Center, Academia Sinica, 128 Academia Road, Section 2, Taipei, 115201 Taiwan; 2 Guangxi Key Laboratory of Plant Conservation and Restoration Ecology in Karst Terrain, Guangxi Institute of Botany, Guangxi Zhuangzu Autonomous Region and Chinese Academy of Sciences, Guilin, 541006 China

**Keywords:** Molecular evolution, Taxonomy, Evolution, Plant sciences, Plant evolution

## Abstract

*Petrocodon* and *Primulina* are two characteristic genera of Gesneriaceae that exhibit remarkable species and floral diversity, and high endemism across the Sino-Vietnamese Limestone Karsts. To better understand the evolution of limestone gesneriad plastomes, we report nine complete plastomes of seven *Primulina* and two *Petrocodon* which have never been assembled before. The newly generated plastomes range from 152,323 to 153,786 bp in size and display a typical quadripartite structure. To further explore the plastome evolution across Gesneriaceae, we assembled five additional plastomes from public reads data and incorporated 38 complete Gesneriaceae plastomes available online into comparative and phylogenomic analyses. The comparison of 52 Gesneriaceae plastomes reveals that not only *Primulina* and *Petrocodon* but all gesneriad genera analyzed are highly conserved in genome size, genome structure, gene contents, IR boundary configurations, and codon usage bias. Additionally, sliding window analyses were implemented across alignments of *Primulina* and *Petrocodon* for identifying highly variable regions, providing informative markers for future studies. Meanwhile, the SSRs and long repeats of Gesneriaceae plastomes were characterized, serving as useful data in studying population and repetitive sequence evolutions. The results of plastome phylogenetics represent a preliminary but highly resolved maternal backbone genealogy of *Primulina* and the Old World subtribes of Gesneriaceae.

Gesneriaceae is a medium-sized plant family comprising more than 3400 species in ca. 150 genera distributed primarily in tropics and subtropics around the world^1,2^. Species of Gesneriaceae inhabit a wide range of habitats and exhibit multifarious evolutionary adaptations^2,3^, resulting in numerous aesthetic plants with high horticultural values^4^. One of the most notable habitats that gesneriads thrive is the limestone karst^5,6^, a distinctive landscape and internal drainage system formed by the dissolution of the highly soluble and porous carbonate sedimentary rock^7,8^. Because of the high salinity and porosity, poorly developed soils, and fragmented landscapes, limestone karsts generally impose hardship on the survival of plants^8^, allowing only those that have evolved to adapt to the limestone bedrock^7,9^. Striding across the bordering areas of southern China and northern Vietnam lies an immense and spectacular landscape^10^ noted as the Sino-Vietnamese Limestone Karsts (SVLK)^7,9^. As one of the largest continuous karsts in the world^10^, SVLK are known for many celebrating tourist destinations and UNESCO World Heritages Sites^11^ of international geomorphological significance. Additionally, SVLK are also major biodiversity hotspots^9,11^ abounding with many species-rich and narrowly distributed endemic plants^9^ such as the two sister gesneriad genera^3^ *Primulina* Hance^6^ and *Petrocodon* Hance^12^.

Prior to the molecular recircumscription, *Primulina* (*Pr.*) was known as a monotypic genus composed solely of *Pr. tabacum* Hance, a prioritized key protected species distributed in southern China^13^. Molecular phylogenetic studies, however, showed that *Pr. tabacum* was nested within a strongly supported clade composed of *Chirita* sect. *Gibbosaccus* Clarke (ca. 120 spp.), *Chiritopsis* W.T.Wang (17 taxa), and two of the three described species of *Wentsaiboea* D.Fang & D.H.Qin that are all mainly distributed in the SVLK^6,14,15^. Based on taxonomic priority, *Primulina* was expanded to include the ca. 140 species of these three genera^14,15^. With more than 70 new species added to the genus after its recircumscription, *Primulina* now contains more than 200 species, representing one of the largest Old World gesneriad genera^5,6^. The high species diversity and restricted distributions on limestone habitats have made the calciphilous *Primulina* an ideal study subject for understanding plant radiation on SVLK^6^. Several studies have employed Sanger sequencing data to reconstruct phylogenetic relationships of *Primulina* for understanding its underlying speciation mechanisms^6,16,17^. Additionally, the genome-scale data of *Primulina* have gradually increased with the advancement of next generation sequencing (NGS) technology. For instance, Ai et al.^18^ sequenced 11 *Primulina* transcriptomes, and Feng et al.^19^ assembled the complete genome of *Pr. huaijiensis* Z.L.Ning & Jing Wang. Furthermore, there are eight plastomes of seven *Primulina* species, i.e., *Pr. brachytricha* var. *magnibracteata* (W.T.Wang & D.Y.Chen) Mich.Möller & A.Weber, *Pr. eburnea* (Hance) Yin Z.Wang, *Pr. huaijiensis*, *Pr. liboensis* (W.T.Wang & D.Y.Chen) Mich.Möller & A.Weber, *Pr. linearifolia* (W.T.Wang) Yin Z.Wang, *Pr. ophiopogoides* (D.Fang & W.T.Wang) Yin Z.Wang, and *Pr. tenuituba* (W.T.Wang) Yin Z.Wang, available on GenBank up to date (Table S1).

Similar to *Primulina*, the circumscription of *Petrocodon* (*Pe.*) also experienced drastic changes as molecular phylogenetic analyses revealed that the originally defined *Petrocodon* is polyphyletic, nested within a strongly supported clade composed of the mono- or oligotypic genera (i.e., *Calcareoboea* C.Y.Wu ex H.W.Li, *Dolicholoma* D.Fang & W.T.Wang, *Lagarosolen* W.T.Wang, *Paralagarosolen* Y.G.Wei, and *Tengia* Chun), and some species of *Didymocarpus* Wall. and *Wentsaiboea* D.Fang & D.H.Qin that are also restricted to SVLK^20^. Comparing to the species-rich *Primulina*, however, *Petrocodon* contains only ca. 50 taxa^5,12^ or a quarter of the species diversity of its sister genus. However, flower morphology of *Petrocodon* is even more variable than *Primulina* in terms of corolla color, symmetry, shapes, and even stamen number^12,20,21^, likely resulting from adaptive evolution triggered by diverse pollination syndromes^21^. However, since all previous phylogenetic studies of *Petrocodon* relied solely on two DNA markers, the nuclear ITS and chloroplast *trnL*^*UAA*^*-trnF*^*GAA*^, its interspecific relationships have yet to be robustly resolved^12,20^. Additionally, in comparison with *Primulina*, the dearth of genomic data in *Petrocodon* is apparent since the only available data to date is the plastome of *Pe. jinxiensis* (Yan Liu, H.S.Gao & W.B.Xu) A.Weber & Mich.Möller (Table S1). As a result, it is necessary to generate more genomic data in order to provide greater insight into the evolution of the extraordinary floral diversity of *Petrocodon*.

Despite thousand copies per organelle, plastid genome (plastome) is overall highly conserved across various land plant lineages^22^. The typical structure of a plastome is represented as a circular molecule consisting of two large rRNA-encoding inverted repeats (IRs) separated by a large single copy (LSC) region and a small single copy (SSC) region, forming a quadripartite structure^22,23^. Most of the land plant plastomes contain about 110–130 genes including eight rRNA genes (four duplicated genes in IRs), 30–35 tRNA genes, and 70–88 protein coding genes, and the genome sizes are usually in the range around 150–160 kb^22^. Though evolving about half slower than nuclear DNAs^22^, plastid DNAs have been essential in plant molecular phylogenetics^24^ for their high copy number, uniparental inheritance, relatively small genome size, and haploidy that are easier to work with and more likely to obviate problems caused by frequent recombination, polyploidy, or paralogues in nuclear phylogenetic analyses^25^. Currently, 8887 plastome sequences of land plants (https://www.ncbi.nlm.nih.gov/genome/browse/#!/organelles/; accessed 25 July 2022) have been deposited in GenBank up to date, mostly assembled using NGS technologies in the recent decade. The boom of data allows more plastomes to be compared and analyzed, facilitating our understanding of plastome evolution and greatly improving phylogenetic resolutions^23,26^.

In this study, we report nine complete plastomes of seven *Primulina* and two *Petrocodon* species, all of which have never been sequenced before (Table 1). In addition, all available Gesneriaceae plastomes on the GenBank were downloaded (accessed in February 1st, 2022) and five whole-genome shotgun (WGS) sequencing data from the Sequence Read Archive (SRA) were retrieved to generate additional plastomes (Table S1). Based on these data, we intend to (1) conduct the comparative analyses of plastome structure, gene content, repeat content, and codon usage bias of Gesneriaceae plastomes to improve our understanding of plastome evolution in the family; (2) identify the variable regions in *Primulina* and *Petrocodon* plastomes for further phylogeographic and phylogenetic studies; (3) reconstruct the phylogenetic relationships using plastome sequences to provide fundamental insights into the phylogenetic relationships of *Primulina* and other Gesneriaceae taxa. Our data and results could serve as valuable resources for both the evolutionary and conservation biology of *Primulina* and *Petrocodon*.

**Table 1:** Statistics of nine newly generated plastomes of *Petrocodon* and *Primulina*.

Species	Voucher specimen	Herbarium accession	Length (bp)	GC content (%)	LSC (bp)	SSC (bp)	IR (bp)	Average coverage	Total reads	Number of reads mapped	% of reads mapped	NCBI accession
*Petrocodon coriaceifolius*	*Chung 2943*	HAST 142670	153,292	37.5	84,345	18,083	25,432	146.4 ± 29.4	1,780,412	84,632	4.75	MZ675547
*Petrocodon multiflorus*	*Chung 2913*	HAST 142641	153,227	37.5	84,118	18,279	25,415	318.3 ± 61.9	3,407,412	188,042	5.52	MZ675548
*Primulina cordata*	*Chung 3021*	HAST 142745	153,786	37.5	84,789	18,085	25,456	60.9 ± 16.5	1,784,254	35,440	1.99	MZ675549
*Primulina fimbrisepala*	*B. Pan s.n*	IBK	153,089	37.5	84,684	17,527	25,439	87.2 ± 21.7	1,754,894	50,342	2.87	MZ675550
*Primulina hezhouensis*	*Chung 2914*	HAST 142642	153,556	37.6	84,757	17,887	25,456	100 ± 26.1	2,156,686	58,386	2.71	MZ675551
*Primulina lutea*	*Chung 2916*	HAST 142644	153,262	37.6	84,094	18,188	25,490	125.5 ± 33.5	3,635,802	74,380	2.05	MZ675552
*Primulina medica*	*Chung 2962*	HAST142642	152,323	37.6	84,890	16,489	25,472	125.9 ± 34.1	1,735,850	72,720	4.19	MZ675553
*Primulina pengii*	*Peng 24024*	HAST 140645	152,354	37.7	83,218	18,146	25,495	190.3 ± 72.3	4,354,548	87,526	2.01	MZ675554
*Primulina sclerophylla*	*Chung 2971*	HAST 142697	153,185	37.6	84,608	17,641	25,468	94.0 ± 21.3	1,876,714	54,416	2.90	MZ675555

*HAST* Herbarium of Biodiversity Research Center, Academia Sinica, Taipei, *IBK* Herbarium of Guangxi Institute of Botany.

## Results

### Features of the newly sequenced *Petrocodon* and *Primulina* plastomes

**Figure 1:**
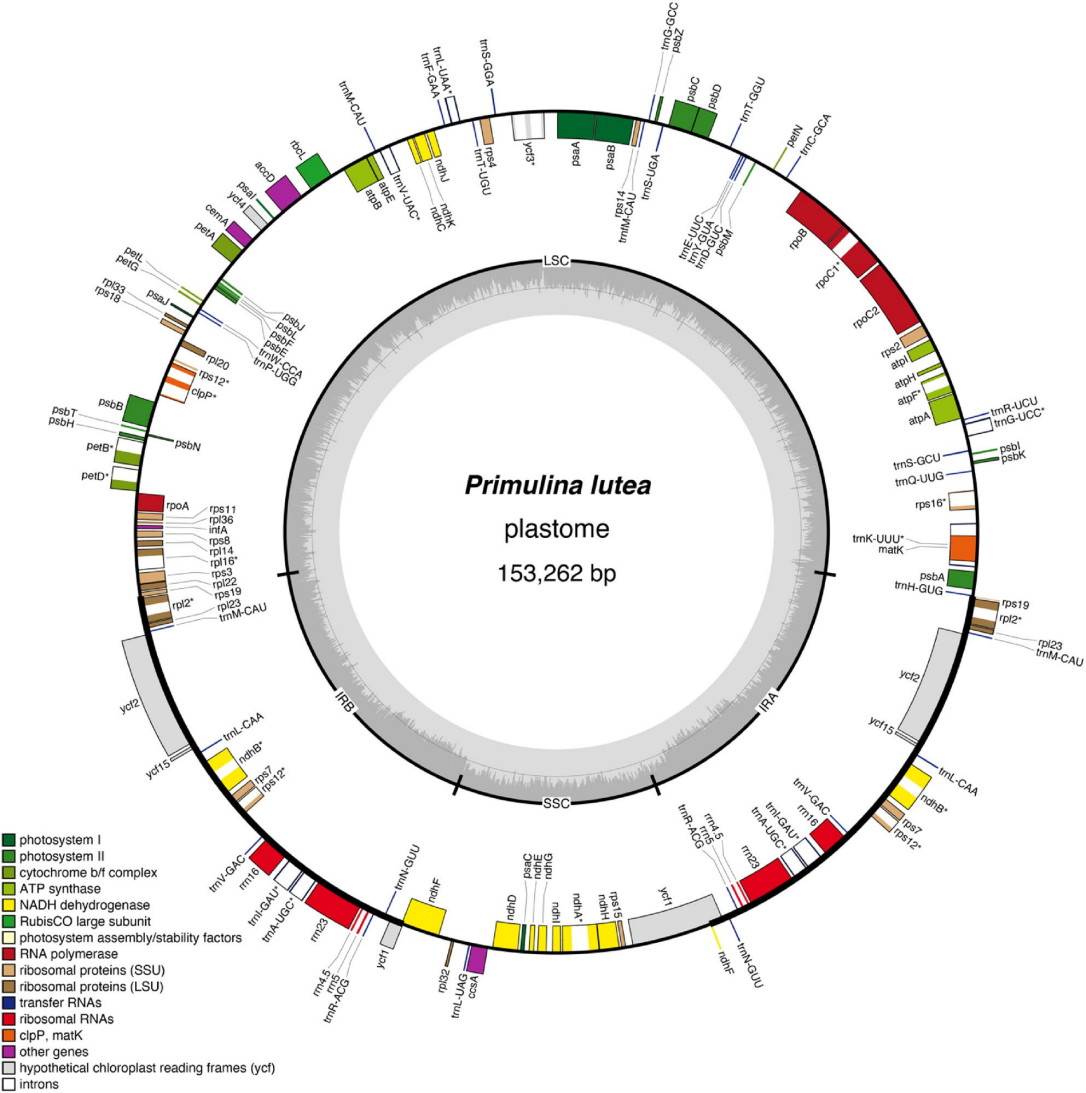
The plastome map of *Primulina lutea*. Genes drawn on the inner side of the outer circle are transcribed clockwise, and those on the outer side are transcribed counterclockwise. IRs are shown in bold line in the outer circle. The inner circle indicates GC contents across the genome with lighter gray indicating AT contents. Genes belonging to different functional groups were shown in different colors. Gene name ends with asterisk (*) indicates the intron containing gene.

Sizes of the nine newly sequenced plastomes of *Petrocodon* and *Primulina* range from 152,323 bp in *Pr. medica* (D.Fang ex W.T.Wang) Yin Z.Wang to 153,786 bp in *Pr. cordata* Mich.Möller & A.Weber (Tables 1, S1). These nine plastomes display the typical quadripartite structure of most angiosperms (Figs. 1, S1–S8), with one LSC (83,218 in *Pr. pengii* W.B.Xu & K.F.Chung to 84,890 bp in *Pr. medica*), one SSC (16,489 in *Pr. medica* to 18,279 bp in *Pe. multiflorus* F.Wen & Y.S.Jiang), and a pair of IRs (25,415 in *Pe. multiflorus* to 25,495 bp in *Pr. pengii*). GC contents of the nine plastomes also show limited variation (37.5–37.7%). For assembly quality, there were 35,440 (*Pr. cordata*) to 188,042 (*Pe. multiflorus*) reads mapped to the nine plastomes whose mean coverages range from 60.9 ± 16.5 × in *Pr. cordata* to 318.3 ± 61.9 × in *Pe. multiflorus* (Table 1). The gene content and order are identical in the nine plastomes (Fig. 1, S1–8). These plastomes contain 114 unique genes, including 80 protein coding genes, 30 tRNA genes, and four rRNA genes, of which 15 genes have one intron (including *rps12*), two genes have two introns, and 17 genes are completely duplicated in IRs. Similar to a majority of angiosperm plastomes^27^, the *rps12* in the nine plastomes is a trans-splicing gene with three exons of which two are duplicated in IRs (Table 2; Fig. 1). In addition, *ndhF*, *rps19*, and *ycf1* are partially duplicated in IRs since these genes are located at IR/SC boundaries (Figs. S9, S10).

**Table 2:** The gene contents of 52 Gesneriaceae plastomes analyzed in this study.

Gene category	Gene name
Photosystem I	*psaA*, *psaB*, *psaC*, *psaI*, *psaJ*
Photosystem II	*psbA*, *psbB*, *psbC*, *psbD*, *psbE*, *psbF*, *psbH*, *psbI*, *psbJ*, *psbK*, *psbL*, *psbM*, *psbN*, *psbT*, *psbZ*
Cytochrome b/f complex	*petA*, petB*, petD*, *petG*, *petL*, *petN*
ATP synthase	*atpA*, *atpB*, *atpE*, atpF*, *atpH*, *atpI*
NADH dehydrogenase	ndhA*, *ndhB*(×2)*, *ndhC*, *ndhD*, *ndhE*, *ndhF#*, *ndhG*, *ndhH*, *ndhI*, *ndhJ*, *ndhK*
RubisCO large subunit	*rbcL*
RNA polymerase	*rpoA*, *rpoB*, rpoC1*, *rpoC2*
Ribosomal proteins (SSU)	*rps2*, *rps3*, *rps4*, *rps7*(×2), *rps8*, *rps11*, *rps12*(×1.67)*, *rps14*^2^, *rps15*, rps16*, *rps18*, *rps19*#^1^
Ribosomal proteins (LSU)	*rpl2*(×2)*, *rpl14*, *rpl16*, *rpl20*, *rpl22*, *rpl23*(×2), *rpl32*, *rpl33*, *rpl36*
Protease, Maturase	clpP**, *matK*
Other genes	*accD*, *ccs1*, *ccsA*, *infA*, *cemA*
Hypothetical chloroplast reading frames (ycf)	*ycf1*#, *ycf2*(×2), ycf3**, *ycf4*, *ycf15*(×2)
Transfer RNAs (tRNA)	*trnA-UGC*(×2)*, *trnC-GCA*, *trnD-GUC*, *trnE-UUC*, *trnF-GAA*, *trnG-GCC*, *trnG-UCC*, *trnH-GUG*, *trnI-GAU*(×2)*, trnK-UUU*, *trnL-CAA*(×2), trnL-UAA*, *trnL-UAG*, *trnM-CAU* (LSC)^3^, *trnM-CAU(*×*2)*(IR)^3^, *trnfM-CAU*, *trnN-GUU*(×2), *trnP-UGG*, *trnQ-UUG*, *trnR-ACG*(×2), *trnR-UCU*, *trnS-GCU*, *trnS-GGA*, *trnS-UGA*, *trnT-GGU*, *trnT-UGU*, *trnV-GAC*(×2), trnV-UAC*, *trnW-CCA*, *trnY-GUA*
Ribosomal RNAs (rRNA)	*rrn4.5*(×2), *rrn5*(×2), *rrn16*(×2), *rrn2*(×2)

(×2): gene duplicated in IRs. *Gene with one intron. **Gene with two introns. (×1.67): the trans-splicing gene, *rps12*, which possesses three exons with two of which duplicated in IRs, and thus the gene was marked as ×1.67 copies. ^#^The partially duplicated genes locating on the IR/SC boundaries. ^1^*Rps19* of some *Paraboea* species is not partially duplicated by IRs. ^2^*Rps14* is loss in *Oreocharis mileensis*. ^3^The *trnM-CAU* gene in LSC is different in sequence from the *trnM-CAU* in IRs, and thus regarded as distinct genes.

### Comparisons of plastome structure and IR boundaries

**Figure 2:**
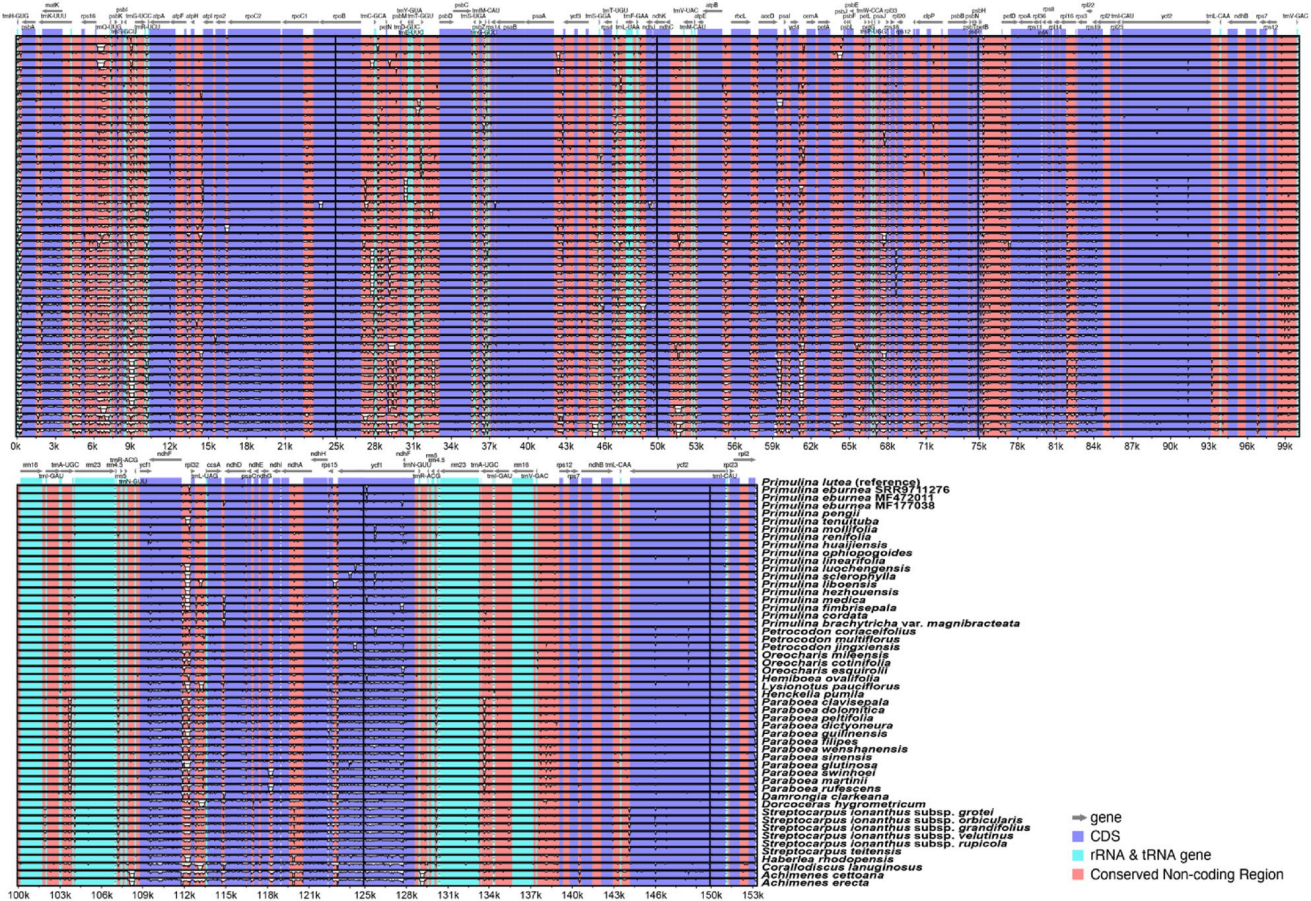
The plastome similarity comparison of Gesneriaceae plastomes using mVISTA. *Primulina lutea* was set as the reference. The filled boxes inside each row indicate the sequence similarities. Similarities lower than 50% are not shown. Purple boxes represent the protein-coding regions, cyan blue boxes represent the RNA genes, and red boxes are intergenic (non-coding) regions.

The comparison of sequence similarity performed by mVISTA^28^ showed that both the structure and sequence content are highly conserved across different gesneriad species and genera (Fig. 2). The IR/SC boundaries and the positions of the genes flanking these boundaries among the Gesneriaceae plastomes analyzed here are highly conserved with no significant boundary shift (Figs. S9, S10). Among all the examined gesneriad plastomes, except for *Haberlea rhodopensis* Friv. and several *Paraboea* (C.B.Clarke) Ridl. species, the boundaries of LSC/IRB, IRB/SSC, SSC/IRA, and IRA/LSC are located within the reading frames of *rps19*, *ndhF*, *ycf1*, and between the *trnH-GUG* and the partially duplicated *rps19*, respectively (Figs. S9, S10). The IRB/SSC boundary of *H. rhodopensis* differs from others since the coding region of its *ndhF* is shorter (2241 bp) than other species (2262–2307 bp), and thus does not extend into IRB (Fig. S9). The LSC/IRB boundary of the eight *Paraboea* species located outside *rps19*, resulting in no partial duplication of *rps19* at the IRA/LSC boundary in those plastomes (Fig. S9).

### SSR and long repeat analyses of Gesneriaceae plastomes

**Figure 3:**
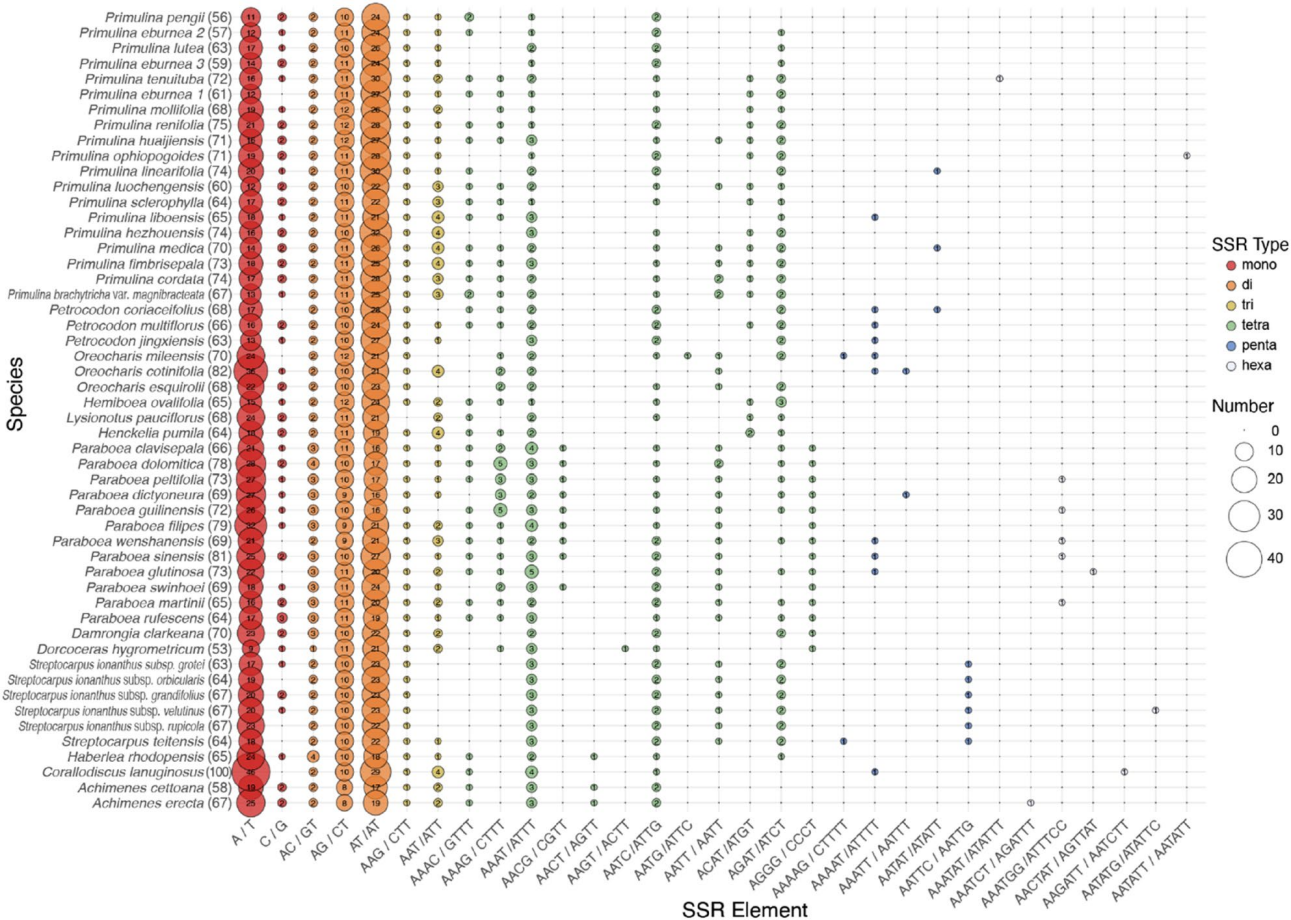
The balloon plot of detailed numbers of each SSR repeat pattern (unit pattern) of 52 Gesneriaceae plastomes with one IR excluded. Total number of SSRs in each plastome is shown in the parentheses after the scientific name. 1: *Pr. eburnea* MF177038. 2: *Pr. eburnea* MF472011. 3: *Pr. eburnea* SRR9711276.

The simple sequence repeats (SSRs) detected by MISA^29^ are listed in Table S2 and their distributions in each species are plotted in Fig. 3. A total of 3551 SSRs were found in the 52 Gesneriaceae plastomes, with the number of SSRs in each sequence ranging from 53 in *Dorcoceras hygrometricum* Bunge to 100 in *Corallodiscus lanuginosus* (Wall. ex R.Br.) B.L.Burtt. Dinucleotide repeats are the most abundant type across all species (totaling 1860 SSRs), followed by mono- (1097), tetra- (424), tri- (136), penta- (23), and hexanucleotide repeats (11). Among 31 different kinds of repeat units identified, AT-rich units are more abundant in terms of both unit types and SSR counts than GC-rich units (Fig. 3). For example, the A/T mononucleotide SSRs are greater in number than G/C mononucleotide SSRs in all samples, while many of the pent- and hexanucleotide repeat units consist exclusively of A/T bases (Fig. 3). Additionally, all of the hexa- and some of the pentanucleotide SSRs are unique to specific species or genera. For instance, AATTC/AATTG is unique to *Streptocarpus* Lindl., and AATATT/AATATT was only found in *Pr. ophiopogoides* (D.Fang & W.T.Wang) Yin Z.Wang (Fig. 3).

**Figure 4:**
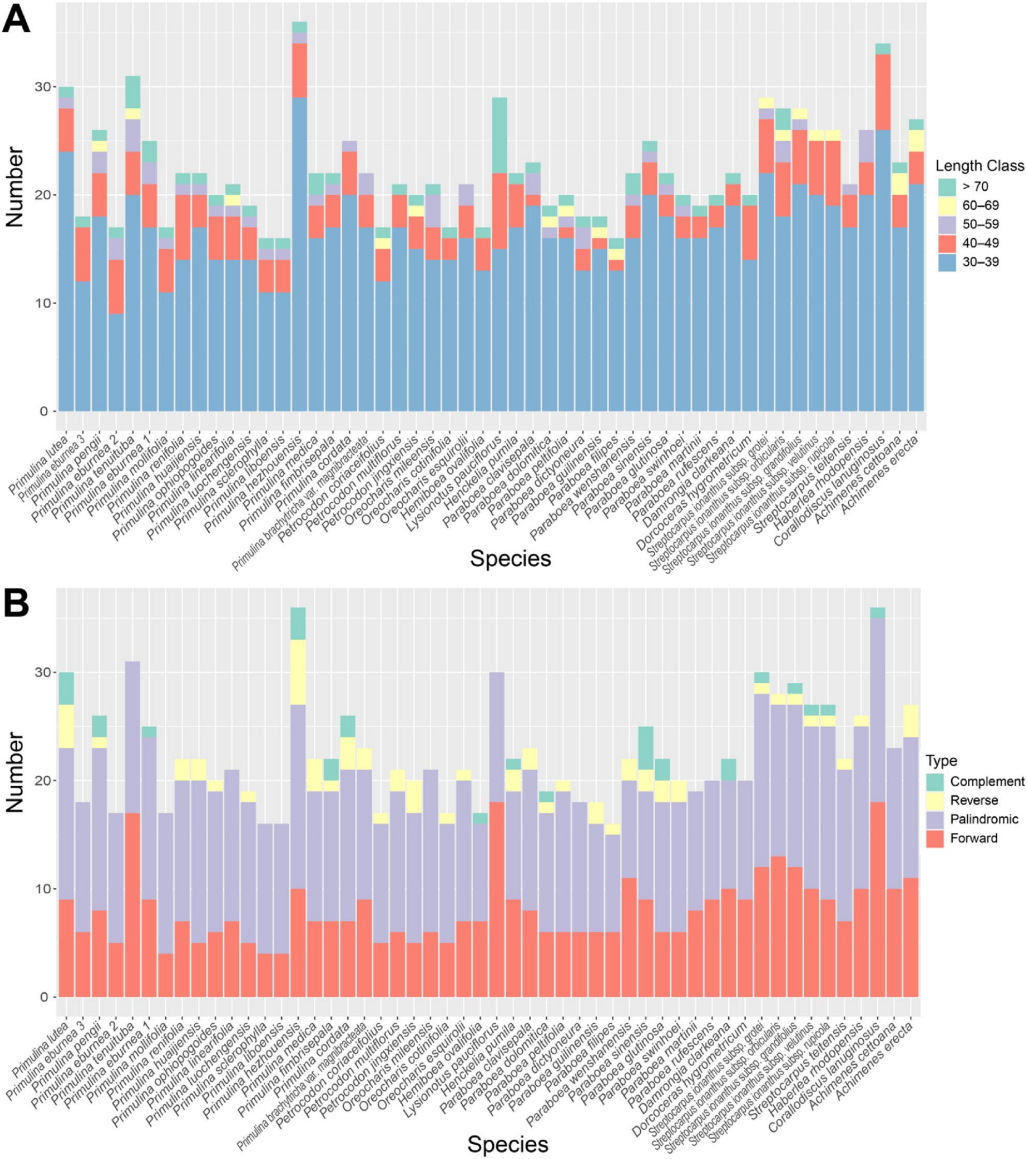
The statistics of long repeat contents in 52 Gesneriaceae plastomes with one IR excluded. (**A**) The length distribution of long repeats in five length classes. (**B**) The number of four types of long repeats in each plastome. ^1^*Pr. eburnea* MF177038. ^2^*Pr. eburnea* MF472011. ^3^*Pr. eburnea* SRR9711276.

The long repeats detected by REPuter^30^ are summarized in Table S3 and Fig. 4. The total number of long repeats in each plastome varies from 27 in *Paraboea filipes* (Hance) B.L.Burtt, *Pr. sclerophylla* (W.T.Wang) Yan Liu, and *Pr. liboensis* to 36 in *Pr. hezhouensis* (W.H.Wu & W.B.Xu) W.B.Xu & K.F.Chung. Among the four repeat types, palindromic repeat is the most abundant type (totaling 664 repeats across all plastomes), followed by forward (540), reverse (41), and complement (19) repeats (Fig. 4B). Most of the repeats fall into the range class of 30–39 bp in length (867 repeats across all plastomes), followed by the range class 40–49 bp (177), ≥ 70 bp (55), 50–59 bp (47), and 60–69 bp (18) (Fig. 4A), while the longest repeat reaches 200 bp found in *Lysionotus pauciflorus* Maxim. (Table S3).

### Codon usage bias in Gesneriaceae

**Figure 5:**
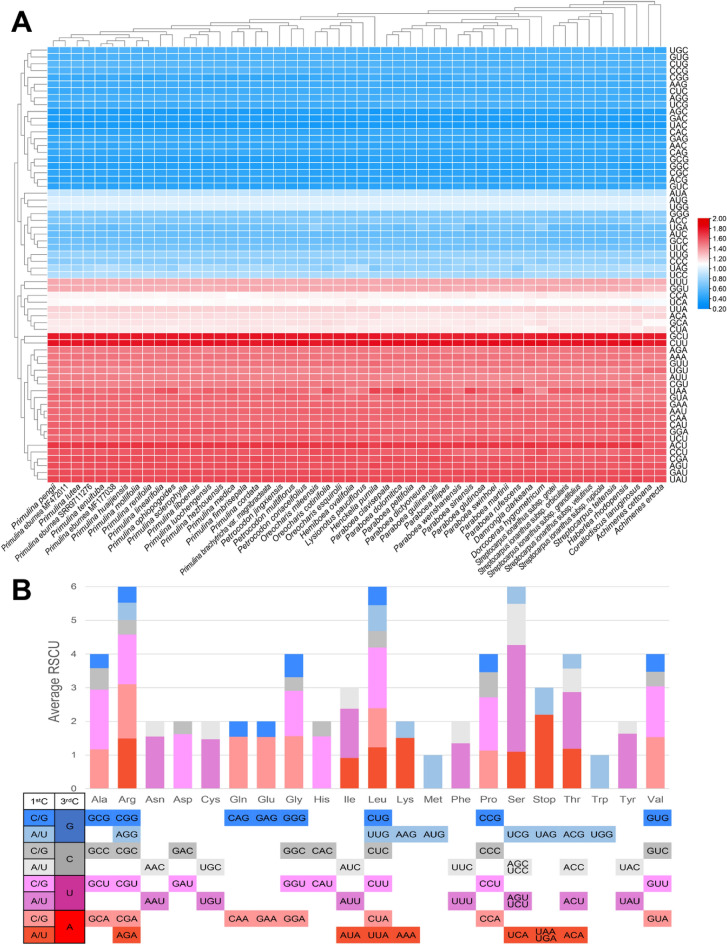
The RSCU of 80 protein-coding genes of 52 Gesneriaceae Plastomes. (**A**) The heat map of RSCU of 63 codons across 52 plastomes. Red boxes indicate highly preferred synonymous codon (RSCU > 1), while the blue boxes imply the less preferred synonymous codon (RSCU < 1). (**B**) The average RSCU of 52 Gesneriaceae plastomes colored by the content of the first (1st C) and the third codon positions (3rd C). ^1^*Pr. eburnea* MF177038. ^2^*Pr. eburnea* MF472011. ^3^*Pr. eburnea* SRR9711276.

The observed codon usage frequencies and relative synonymous codon usage (RSCU) values of 80 protein-coding genes for each of the 52 Gesneriaceae plastomes evaluated by DAMBE v.7.3.2^31^ are provided in Table S4 and the average RSCU values were summarized in Fig. 5. Of the observed codon usage frequency, the codon AUU encoding isoleucine is the most frequent codon (a total of 50,164 counts), while the stop codon UGA is the least frequent (892) counts (Table S4). The comparison of RSCU values across each sample demonstrates that the codon usage pattern is highly conserved across Gesneriaceae (Fig. 5A) and is biased toward A or T, especially at the third codon position of synonymous codons (Fig. 5). For instance, every sample has the same set of 30 highly preferred codons (RSCU > 1), two codons without preferential bias (since there is no synonymous codon for UGG and AUG; RSCU = 1), and 32 less preferred codons (RSCU < 1), while there is no codon terminated in C or G among the 30 highly preferred codons (Table S4; Fig. 5).

### Sequence divergence analyses

**Figure 6:**
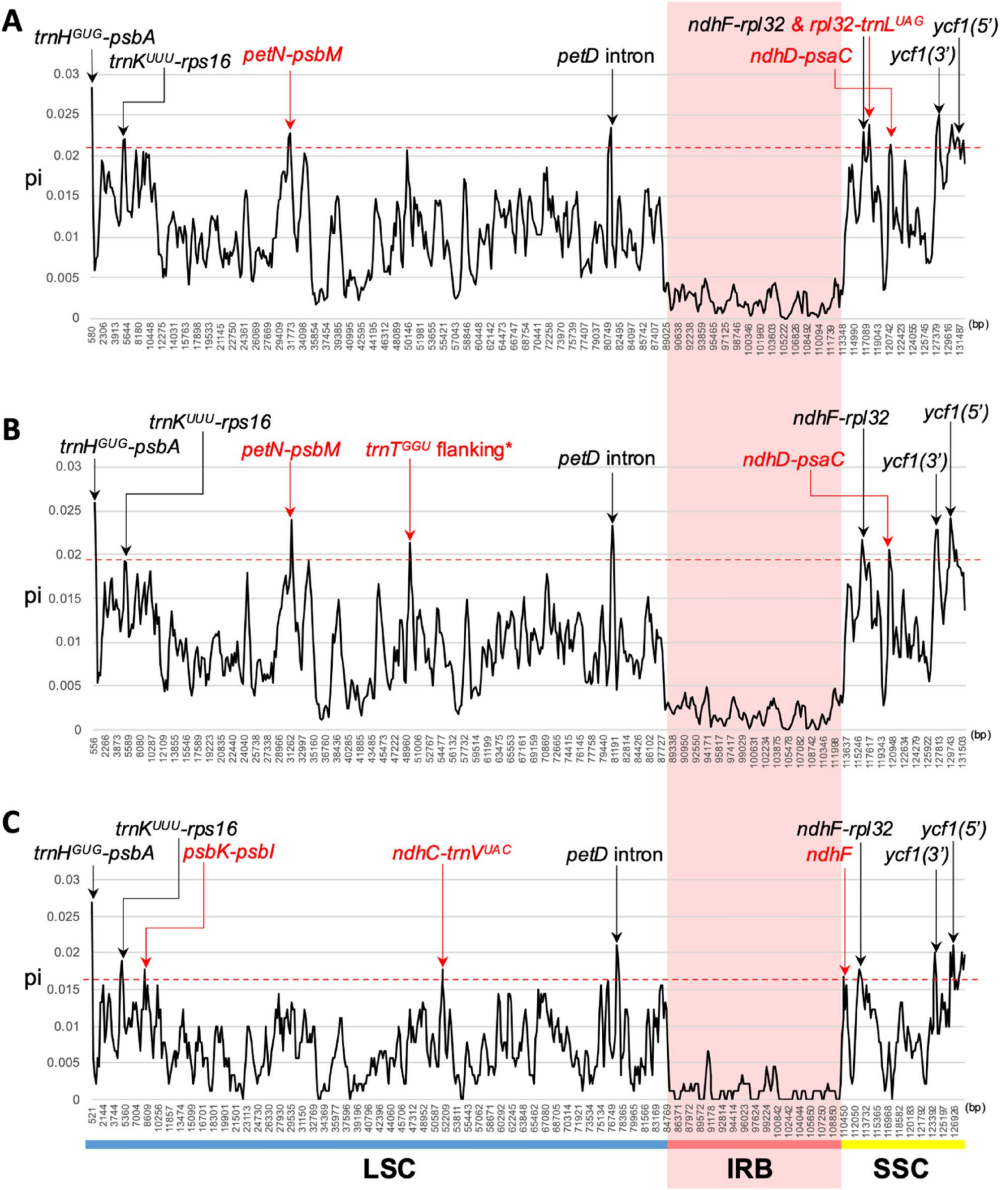
The nucleotide diversity among 19 *Primulina* and three *Petrocodon* plastomes. (**A**) The sliding window analysis across the alignment of 19 *Primulina* + three *Petrocodon* accessions. (**B**) The sliding window analysis across the alignment of 19 *Primulina* plastomes. (**C**) The sliding window analysis across the alignment of three *Petrocodon* plastomes. The red dashed line indicates the lowest *pi* value of 95th percentile windows in the specific alignment. Regions whose *pi* values are higher than the red dashed line were marked as variable regions. Names of the variable regions colored in black indicate that they are universal to three alignments, while those colored in red indicate they were identified only in one or two of the alignment(s). *The flanking regions of *trnT-GGU*.

To detect variable regions in *Primulina* and *Petrocodon*, we conducted sliding window analyses for three alignments, *Primulina* (19 sequences, 132,094 bp), *Petrocodon* (three sequences, 128,477 bp), and *Primulina* + *Petrocodon* (22 sequences, 132,546 bp), and identified variable regions for each alignment based on the windows with 95th percentile *pi* values using DnaSP v.6.12.03^32^. In the alignment of *Primulina* + *Petrocodon*, six intergenic spacers (*trnH*^*GUG*^*-psbA*, *trnK*^*UUU*^*-rps16*, *petN-psbM*, *ndhF-rpl32*, *rpl32-trnL*^*UAG*^, and *ndhD-psaC*), *petD* intron, and two coding regions in the *ycf1*, i.e., *ycf1(3′)* closer to the 3′ end of the reading frame and *ycf1(5*′*)* closer to the 5′ end of the gene, were detected as highly variable regions (Fig. 6A). In the alignment containing only *Primulina*, five intergenic spacers (*trnH*^*GUG*^*-psbA*, *trnK*^*UUU*^*-rps16*, *petN-psbM*, *ndhF-rpl32*, and *ndhD-psaC*), flanking regions around *trnT-GGU*, *petD* intron, and CDS of *ycf1(3*′*)* and *ycf1(5*′*)* were considered as highly variable regions (Fig. 6B). In the alignment of three *Petrocodon* plastomes, we identified five intergenic spacers (*trnH*^*GUG*^*-psbA*, *trnK*^*UUU*^*-rps16*, *psbK-psbI*, *ndhC-trnV*^*UAC*^, and *ndhF-rpl32*), *petD* intron, and *ndhF*, *ycf3(3*′*)*, and *ycf1(5*′*)* CDS as potential phylogenetic markers for the genus (Fig. 6C). Six regions, i.e., *trnH*^*GUG*^*-psbA*, *trnK*^*UUU*^*-rps16*, *petD* intron, *ndhF-rpl32*, *ycf1(3*′*)*, and *ycf1(5*′*)*, were detected as most variable in all of the three analyses, while *trnT-GGU* flanking regions was recognized exclusively in *Primulina*, *psbK-psbI*, *trnC-trnV* and *ndhF* CDS exclusively in *Petrocodon*, and *petN-psbM* and *ndhD-psaC* in both *Primulina* and *Primulina* + *Petrocodon* alignments (Fig. 6). In addition, there is no variable region detected in IR since its *pi* values are generally lower than the SC regions (Fig. 6).

### Phylogenetic analyses

**Figure 7:**
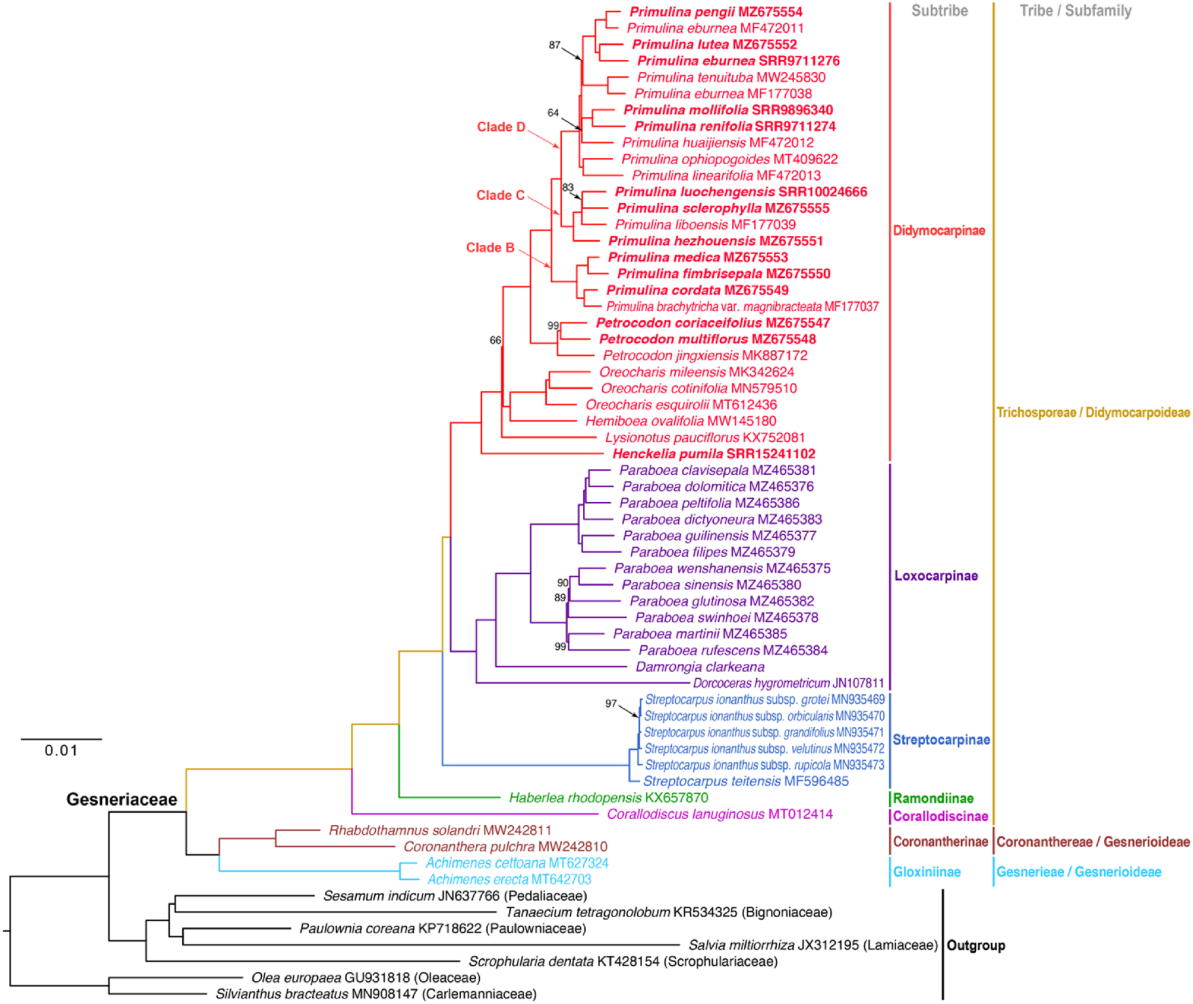
The ML phylogram of 54 Gesneriaceae plastomes and seven Lamiales outgroups reconstructed by IQ-TREE. Nodes with full ultrafast bootstrap support values were not labeled with any number, while those values which are not equal to 100 were labeled out. Each clade within Gesneriaceae was colored by subtribes following the classification in Weber et al.^1^. The three major clades of *Primulina* were labeled corresponding to the Clades B, C, and D in Kong et al.^16^. Species name in bold indicates that the plastome is generated or assembled in this study.

The partitioned alignment of 61 plastomes with one IR excluded is 153,963 bp in length, of which 41,572 (30.5% of the total length) were variable and 20,830 (15.3%) were parsimoniously informative. The substitution models and best-fit partition schemes estimated by ModelFinder^33^ for IQ-TREE^34^ and MrBayes^35^ are summarized in Table S5. Phylogenetic relationships reconstructed by the three phylogenetic programs RAxML^36^, IQ-TREE^34^, and MrBayes^35^ are all highly supported and congruent except for the position of *Pr. huaijiensis* that varied slightly among different analyses (Figs. 7, S11, S12). Specifically, *Pr. huaijiensis* is sister to *Pr. mollifolia* (D.Fang & W.T.Wang) J.M.Li & Yin Z.Wang + *Pr. renifolia* (D.Fang & D.H.Qin) J.M.Li & Yin Z.Wang with poor support value (ultrafast bootstrap support = 66) in the IQ-TREE phylogram (Fig. 7), while the species is sister to *Pr. tenuituba* + *Pr. eburnea* (MF177038) with low supports (bootstrap value/posterior probability = 48/0.9994) in the trees computed by RAxML (Fig. S11) and MrBayes (Fig. S12), respectively.

As depicted in Fig. 7, monophylies of tribes, subtribe, and genera circumscribed by Weber et al.^1^ are fully supported and relationships among these taxa are all well resolved (Figs. 7, S11, S12). Specifically, the predominantly New World subfamily Gesnerioideae is monophyletic and sister to the monophyletic and mainly Old World subfamily Didymocarpoideae. Within Gesnerioideae, *Achimenes* Pers. (subtribe Gloxiniinae, tribe Gesnerieae) is sister to *Rhabdothamnus* A.Cunn. + *Coronanthera* Vieill. ex C.B.Clarke of the subtribe Coronantherinae, tribe Coronanthereae. Within the subfamily Didymocarpoideae (only tribe Trichiosporeae sampled), subtribes Loxocarpinae and Didymocarpinae form a clade, with subtribes Streptocarpinae (*Streptocarpus* Lindl.), Ramondiinae (*Haberlea*), and Corallodiscinae (*Corallodiscus*) forming successive sister groups to Loxocarpinae + Didymocarpinae. Within subtribe Didymocarpinae, *Lysionotus* and *Henckelia* form successive sister groups to a poorly supported clade (ultrafast bootstrap value of IQ-TREE/bootstrap value of RAxML/posterior probability of MrBayes = 66/77/1.00) composed of two clades, *Petrocodon* + *Primulina* and *Oreocharis* Benth. + *Hemiboea* C.B.Clarke (Figs. 7, S11, S12).

Within *Primulina*, three major clades corresponding to Clades B, C, and D designated in Kong et al.^16^ are also consistently resolved in all three analyses with full support values (Figs. 7, S11, S12). Notably, the three accessions of *Pr. eburnea* are not clustered together. Instead, MF177038, MF472011, and SRR9711276 are sister to *Pr. tenuituba*, *Pr. pengii*, and *Pr. lutea* (Yan Liu & Y.G.Wei) Mich.Möller & A.Weber, respectively (Figs. 7, S11, S12). In *Petrocodon*, *Pe. jingxiensis* is sister to the remaining two species. In *Oreocharis*, *O. cotinifolia* (W.T.Wang) Mich.Möller & A.Weber and *O. mileensis* (W.T.Wang) Mich.Möller & A.Weber, which previously belonged to two different genera^5^, are clustered with *O. esquirolii* H.Lév. (Fig. 7). In *Paraboea*, the species sampled here form two major clades (Figs. 7, S11, S12), consistent with Xu et al.^37^.

## Discussion

### Highly conserved Gesneriaceae plastomes

Our analyses indicate that the genome size, structure, gene content, and IR boundaries of all 52 Gesneriaceae plastomes investigated are highly conserved. Except for a slightly larger outlier of 156,614 bp in *Corallodiscus lanuginosus* (Wall. ex R.Br.) B.L.Burtt, the size of our sampled Gesneriaceae plastomes all falls within the range between 152,323 in *Pr. medica* to 154,245 bp in *Paraboea wenshanensis* Xin Hong & F.Wen (Table 1, S1), close to the median plastome size (154,953 bp) of angiosperms^38^. Gene contents of all 52 gesneriad plastomes are also highly conserved, with only an 83-bp-deletion detected near the 5′-terminal of the reading frame of *rps14* in *Oreocharis mileensis* (W.T.Wang) Mich.Möller & A.Weber (≡ *Paraisometrum mileense* W.T.Wang) that might affect the function of the gene, though this likely case of gene deletion was neglected in the original publication^39^. Compared to the dynamic IR/SC boundaries in plastomes of other Lamiales taxa such as *Amphilophium* Kunth of Bignoniaceae^40^, the configurations around IR/SC boundaries of the 52 gesneriad plastomes are highly conserved (Figs. 2, S9, S10), with the minor variation only detected in *Haberlea rhodopensis* (*ndhF* is not included in IR) and some species of *Paraboea* species (LSC/IRB is located at *rps19-rpl2* spacer) (Fig. S9). No conspicuous boundary shift was detected in the plastomes examined here (Figs. S9, S10), and no genome rearrangement was identified either (Fig. 2).

Comparative plastome analyses across diverse plant lineages such as cupressophytes^41^, Fabaceae^42^, Geraniaceae^43^, *Asarum* L. (Aristolochiaceae)^44^, and *Trachelium* L. (Camapnulaceae)^45^ revealed a positive correlation between the number of repeats and the extent of plastome rearrangement, suggesting a general trend of the repeat-mediated recombination that could have resulted in plastome structural variation^42^. Although plastomes of some species such as the hot pepper *Capsicum annuum* L. possess a considerable number of repetitive sequences and yet are structurally highly conserved^46^, the relatively low number of repeats (Fig. 4) and highly conserved nature of plastomes of Gesneriaceae reported in current study appear to conform well to the expectation of the repeat-mediate recombination hypothesis.

### Codon usage bias in Gesneriaceae plastomes

The redundancy of 64 codons encoding only 20 amino acid and one stop signal, together with factors such as selections and mutational biases, inevitably leads to codon usage bias, and this phenomenon is ubiquitous and variable among different organisms or even within genomes^47^. In angiosperm plastomes, the usage of codons usually biases toward codons with high AT content, especially those synonymous codons with the third position in A or T^48^. This biased tendency is consistent with our results of RSCU analysis (Table S4; Fig. 5). Furthermore, we found that all of the Gesneriaceae plastomes analyzed exhibit highly conserved codon usage patterns (Fig. 5A). Since synonymous codon usage might be associated with various genetic events, e.g., natural selection, mutation, genetic drift^49^, our results of RSCU analysis and the discovery of conserved usage pattern provide preliminary information for better understanding of plastid gene expressions and plastome evolution of Gesneriaceae.

### Potential phylogenetic markers for *Primulina*, *Petrocodon*, and gesneriads

Despite recent advancements in high throughput sequencing technologies, generating and analyzing large-scale plastome data for species-rich genera with more than 200 species such as *Primulina* or population samplings remains a costly and time- and labor-consuming task^50,51^. For efficient species identifications, taxonomic revisions, and inter- and intraspecific studies, it is still crucial to develop phylogenetic informative makers^23^. Since the hypervariable regions are often lineage-specific even among close-related groups^51^, we performed sliding window analyses on three alignments, i.e., *Primulina*, *Petrocodon*, and *Primulina* + *Petrocodon*. Thirteen different variable regions were identified across the three datasets, and six of them are constantly identified in all three alignments (Fig. 6). Amongst the 13 hypervariable regions, four (i.e., *trnH*^*GUG*^*-psbA*, *rpl32-trnL*^*UAG*^, *ycf1_1*, and *ycf1_2*) have been employed for phylogenetic studies of *Primulina*^16,17,52,53^, suggesting the potential of the other nine markers in improving the phylogenetic resolution of *Primulina*. However, compared to the top-9-ranked intrageneric variable regions identified in Feng et al.^50^, only three regions (i.e., *trnH*^*GUG*^*-psbA*, *ndhF-rpl32*, *rpl32-trnL*^*UAG*^) are common in the former and current studies, indicating that variable markers are indeed lineage specific. Nevertheless, the hypervariable regions identified by current study should greatly facilitate systematic and evolutionary research of *Primulina* and especially *Petrocodon* considering all previous studies of the latter genus^12,20^ employed only *trnL*^*UAA*^*-trnF*^*GAA*^ that is slow-evolving and phylogenetically less informative^24^.

Additionally, the plastid SSRs identified in current study (Table S2) provides further molecular markers for population genetic studies of Gesneriaceae. Although contents of SSRs vary across different plastomes in our results, the dominance of A/T mononucleotide SSRs and the strongly AT-biased unit types (Fig. 3) are consistent with the general trend in angiosperm plastomes^54^. Of the 52 gesneriad species investigated, the plastome of *Corallodiscus lanuginosus* possesses the highest number of SSRs (100 repeats) and the second highest number of long repeats (34 repeats, Fig. 4), and these features might have contributed to its larger plastome size (156,614 bp). Interestingly, *C. lanuginosus* is known as a highly variable species with ca. 30 synonyms and distributed widely across remarkable altitudinal, latitudinal, and longitudinal gradients^55^. Zhou et al.^55^ proposed that the wide niche and morphological diversity of *C. lanuginosus* complex might be the consequence of its old divergence in Himalayan-Hengduan Mountains and hybridization/introgression from secondary contacts among populations. Comparing to the low numbers of repetitive sequences and SSRs in plastomes and relatively narrow distribution ranges of a majority of gesneriad species, the abundance of plastid repetitive elements in *C. lanuginosus* might have also been crucial in contributing its intraspecific variation and adaptation to diverse habitats^55^.

### Plastome phylogenomics of Gesneriaceae

In this study, highly supported phylogenies of 54 Gesneriaceae plastomes rooted by seven Lamiales outgroups (Fig. 7) were reconstructed. Of the 19 *Primulina* accessions analyzed, our results are generally congruent with the grouping in Kong et al.^16^ as well as intrageneric relationships inferred by several studies with larger sampling using ITS and plastid markers^6,16,17,53^ with a few exceptions. One notable difference is the position of *Pr. brachytricha* var. *magnibracteata*. In Sanger sequencing-based phylogenetic analyses, *Pr. brachytricha* (W.T.Wang & D.Y.Chen) R.B.Mao & Yin Z.Wang var. *brachytricha* was placed in Clade D^16,17^ (equivalent to Clade A in Guo et al.^53^), while *Pr. brachytricha* var. *magnibracteata* was grouped in Clade B (Fig. 7) in current study, indicating that *Pr. brachytricha* var. *brachytricha* and *Pr. brachytricha* var. *magnibracteata* are distantly related. Recent phylogenetic studies have shown that several intraspecific varieties of *Primulina*, e.g., *Pr. repanda* var. *guilinensis* (W.T.Wang) Mich.Möller & A.Weber and *Pr. glandulosa* var. *yangshuoensis* (F.Wen, Yue Wang & Q.X.Zhang) Mich.Möller & A.Weber, were polyphyletic, necessitating nomenclatural changes to restore the monophyly of these taxa^6^. The likely polyphyletic nature of *Pr. brachytricha* urges further taxonomic study. On the other hand, while all three sampled *Pr. eburnea* are all placed in Clade D, they do not form a monophyletic clade (Fig. 7). *Primulina eburnea* is a species complex with a wide geographic range and morphological variation^52,56^ and the non-monophyly of the species has been noted in a previous study^6^. Zhang et al.^57^ reported hybrid individuals between *Pr. eburnea* and the sympatrically distributed *Pr. mabaensis* K.F.Chung & W.B.Xu, suggesting that hybridization could have contributed to the non-monophyly of the three plastomes of *Pr. eburnea* in current study, though further investigation is needed to test alternative explanation such as incomplete lineage sorting^52,56^.

Although our sampling of Gesneriaceae is far from comprehensive, phylogenomic relationships based on plastome sequences are highly congruent with current infrafamilial classification^1^ and relationships among subfamilies, tribes, and subtribes are also largely congruent with phylogenies inferred from the supermatrix of 26 PCR markers of 768 gesneriad species^3^ and a recent study using target enrichment^2^ capturing 418 genes of 78 taxa except for relationships among a few subtribes in tribe Trichosporeae. Specifically, our plastome trees placed Loxocarpinae and Didymocarpinae in one clade sister to Streptocarpinae (Figs. 7, S11, S12), while in Roalson and Roberts^3^ the clade of Streptocarpinae + Loxocarpinae is successively sister to Didissandrinae and Didymocarpinae, and in Ogutcen et al.^2^ the clade of Didymocarpinae + Streptocarpinae is sister to the clade of Loxocarpinae + Didissandrinae. These discordances could have resulted from sampling issues, hybridization/introgression, chloroplast capture, or incomplete lineage sorting^26^, and thus more studies leveraging informative genomic data with comprehensive sampling are needed to explore the extent and causations of the discordance across Gesneriaceae.

## Material and methods

### Taxon sampling

Two species of *Petrocodon* and six species of *Primulina* were collected from Guangxi and Hunan, China, and *Pr. fimbrisepala* was sampled from the botanical garden of Guangxi Institute of Botany, Guangxi, China (Tables 1 and S1). Voucher specimens were deposited in the Herbarium of Biodiversity Research Center, Academia Sinica, Taipei (HAST) and the Herbarium of Guangxi Institute of Botany (IBK). All species were identified by the second author (W.-B.X.), a leading authority in the taxonomy of Chinese Gesneriaceae^5,6,12,21,37,53^. Since all sampled species are not included in the “Threatened Species List of China’s Higher Plants”^13^ and were not collected from protected areas, no permission was required for sampling. All experiments, including the collection of plant materials, were complying with the relevant institutional, national, and international guidelines and legislation. In addition to the newly sequenced species, we downloaded all the 38 complete and two incomplete Gesneriaceae plastomes available on the GenBank, and assembled five additional plastomes from the WGS raw read data of four *Primulina* and one *Henckelia* species from the SRA database (accessed on February 1st, 2022). Consequently, a total of 52 plastomes from 13 Gesneriaceae genera, i.e., *Achimenes*, *Corallodiscus*, *Damrongia*, *Dorcoceras*, *Haberlea*, *Hemiboea*, *Henckelia*, *Lysionotus*, *Oreocharis*, *Paraboea*, *Petrocodon*, *Primulina*, and *Streptocarpus*, were included in our plastome comparative analyses, and 54 sequences of 15 Gesneriaceae genera (with *Coronanthera* and *Rhabdothamnus* included) were sampled for phylogenetic analyses. Overall, 19 *Primulina* (17 species) and three *Petrocodon* (three species) plastomes were analyzed in this study. Following the names used in Xu et al.^5^ and Plants of the World Online (http://plantsoftheworldonline.org/), the species names and accession numbers of the plastomes and raw reads sampled in this study are summarized in Table S1, regardless the names used in the original publication.

### DNA extraction and next generation sequencing

Total genomic DNAs were extracted from 0.01 g of silica gel dried leaves using the modified CTAB method^53^. The quantity and quality of extracted DNAs were assessed by 1% gel electrophoresis, Nanodrop 2000 (Thermo Scientific Inc., Carlsbad, CA, USA), and Qubit Fluorometer (Thermo Scientific). The DNAs passing were sent to High Throughput Genomics Core at Biodiversity Research Center, Academia Sinica, Taipei, Taiwan to conduct library preparation using TruSeq Nano DNA Library Prep kit (Illumina Inc., San Diego, CA, USA) following manufacturer’s instructions with insert size 550 bp in length. The DNA libraries were sequenced using Illumina MiSeq System (Illumina) in pair-end mode with read length of 300 bp.

### Plastome assembly and annotation

The WGS raw reads data of four *Primulina* and one *Henckelia* species were retrieved via NCBI SRA Toolkits v.2.1.11 for plastome assembly. The sequencing quality of both the newly sequenced and downloaded raw reads were evaluated via FastQC v.0.11.5^58^. Low quality portions of the reads were trimmed and filtered by Trimmomatic v.0.36^59^ with the setting of “LEADING:25 TRAILING:25 SLIDINGWINDOW:4:20 MINLEN:200” for the nine newly sequenced samples and customized settings for the five downloaded samples of different read lengths. The trimmed reads were de novo assembled by GetOrganelle v.1.7.5.1^60^ with the settings of “-R 15 -w 0.9 -k 55,85,105,127,131,143,151,171 -F embplant_pt --reduce-reads-for-coverage inf”. The resulting complete sequences or incomplete contigs were checked and finalized by mapping reads to respective sequences using “Map to Reference” function with “Medium–Low Sensitivity/Fast” settings in Geneious Prime v.2021.1.1^61^.

All newly assembled plastomes were annotated based on the annotations of *Arabidopsis thaliana* (L.) Heynh. (GenBank accession: AP000423) using the “Map to Reference” and “Transfer Annotation” function under Geneious. The start and stop codons of each protein-coding gene were manually checked and adjusted. Additionally, length and anti-codon name of each tRNA gene were further verified by searching the gene sequence against the database of tRNAscan-SE web server^62^. Annotated genomes were visualized by OGDRAW v.1.3.1^63^.

### Comparative analyses of Gesneriaceae plastomes and evaluation of sequence divergence in *Primulina* and *Petrocodon*

To compare genome structures and sequence similarities among the 52 complete Gesneriaceae plastomes, sequences were aligned by Shuffle-LAGAN^64^, which could detect rearrangement, and analyzed using mVISTA web-interface^28^. To compare sequence variations across *Primulina* and *Petrocodon*, three alignments (19 *Primulina*, three *Petrocodon*, and 19 *Primulina* + three *Petrocodon*) were analyzed by sliding window computation of nucleotide diversity (*pi*) with the window size of 600 bp and step size of 200 bp in DnaSP v.6.12.03^32^. The regions with the 95th percentile windows were considered highly variable.

### Simple sequence repeats and long repeats characterization of Gesneriaceae plastomes

To characterize SSR contents in plastomes, we utilized MISA^29^ to identify SSRs in all Gesneriaceae plastomes with one IR excluded and analyzed with the threshold of ten repeat units for mono-, five for di-, four for tri-, and three repeat units for tetra-, penta-, and hexanucleotide SSRs. Compound SSRs were detected by setting the interval up to 100 bp in length. The results were visualized in balloon plot generated in R v.4.0.1 using ‘reshape’ and ‘ggplot2’ packages^65^. For the detection of long repeats, REPuter^30^ was employed to search four repeat categories, i.e., forward (direct), reverse, complement, and palindromic (reverse complement) repeats with the minimum repeat size of 30 bp and ≥ 90% repeat identity by setting Hamming distance of 3.

### Codon usage analysis of Gesneriaceae plastomes

For each Gesneriaceae plastome, the protein coding sequences (CDS) of all the 80 unique protein coding genes (Table 2) were extracted under Geneious. The codon usage frequencies and RSCU values of CDS in each plastome were evaluated in DAMBE v.7.3.2^31^ with the first codon excluded in the calculation. The results of RSCU were visualized in heatmap using TBtools v.1.09854^66^.

### Phylogenetic analyses of Gesneriaceae plastomes

In addition to the nine newly sequenced plastomes, we incorporated all the 38 complete and two incomplete Gesneriaceae plastomes available on Genbank and five plastomes assembled from released WGS reads data of Gesneriaceae species into our phylogenetic analyses. For the outgroup, due to the lack of available plastomes from the two most close-related families, Calceolariaceae and Peltantheraceae, plastomes from seven other Lamiales families were sampled (Table S1). A total of 61 plastomes with one IR excluded were aligned by MAFFT v.7450^67^ under Geneious with default settings and were further partitioned into six parts, i.e., CDS codon position 1 (CDS1), CDS codon position 2 (CDS2), CDS codon position 3 (CDS3), introns, tRNA + rRNA genes (RNA), and intergenic spacers. The phylogenetic relationships were reconstructed by both maximum likelihood (ML) and Bayesian inference (BI) methods implemented in RAxML v.8.2.12^36^ and IQ-TREE v.2.0.6^34^, and MrBayes v.3.2.7^35^, respectively. For ML in RAxML, we executed the rapid bootstrap (BS) analysis with “autoFC” bootstrapping criterion and searched for the best-scoring ML tree based on the GTR + GAMMA nucleotide substitution model on the partitioned dataset. In IQ-TREE, the automatic model selection and the best-fit partitioning scheme search were conducted by ModelFinder^33^, and the ML tree reconstruction was carried out using “-m MFP + MERGE” option with 5000 replicates of ultrafast bootstrap (UFBS) approximation^68^. For BI, prior to MrBayes analyses, the best models fitting into MrBayes and the best-fit partition scheme of the partitioned dataset were evaluated by ModelFinder in IQ-TREE using “-m TESTMERGEONLY -mset mrbayes” options. In MrBayes, the priors were set according to the results of IQ-TREE with models unlinked among each partition, and the Marko chain Monte Carlo (MCMC) simulation of 2,500,000 generations was conducted in two independent runs with four chains each and sampling frequency of every 1000 generations. The first 25% of sampled trees were discarded as burn-in, and the remaining trees were summarized into a phylogram with clade credibility (posterior probability) values. The final phylogram from each analysis was visualized by FigTree v.1.4.4 (http://tree.bio.ed.ac.uk/software/figtree/).

## Supplementary Information

Supplementary Information 1.

Supplementary Table S1.

Supplementary Table S2.

Supplementary Table S3.

Supplementary Table S4.

## Data Availability

The newly assembled plastome sequences of *Petrocodon* and *Primulina* have been submitted to NCBI (https://www.ncbi.nlm.nih.gov/) under the accession numbers MZ675547–MZ675555.
