# Supplementary material for: Plastomes of limestone karst gesneriad genera Petrocodon and Primulina, and the comparative plastid phylogenomics of Gesneriaceae
Source: Sci Rep. 2022 Sep 22;12:15800. doi: 10.1038/s41598-022-19812-2 (PMC9500069; doi:10.1038/s41598-022-19812-2)
Supplement: Supplementary file 1 — Supplementary Information 1. [file 41598_2022_19812_MOESM1_ESM.pdf]

Supplementary Material

Plastomes of limestone karst gesneriad genera *Petrocodon* and *Primulina*, and the comparative plastid phylogenomics of Gesneriaceae

Chia-Lun Hsieh[1], Wei-Bin Xu[2] & Kuo-Fang Chung[1*]

[1]Research Museum and Herbarium (HAST), Biodiversity Research Center, Academia Sinica, 128 Academia Road, Section 2, Taipei 115201, Taiwan.

[2]Guangxi Key Laboratory of Plant Conservation and Restoration Ecology in Karst Terrain, Guangxi Institute of Botany, Guangxi Zhuangzu Autonomous Region and Chinese Academy of Sciences, Guilin 541006, China.

*Corresponding author: bochung@gate.sinica.edu.tw

ORCiD: Chia-Lun Hsieh (0000-0002-3342-3654)
Wen-Bin Xu (0000-0002-5602-8753)
Kuo-Fang Chung (0000-0003-3628-2567)

Supplementary Figures

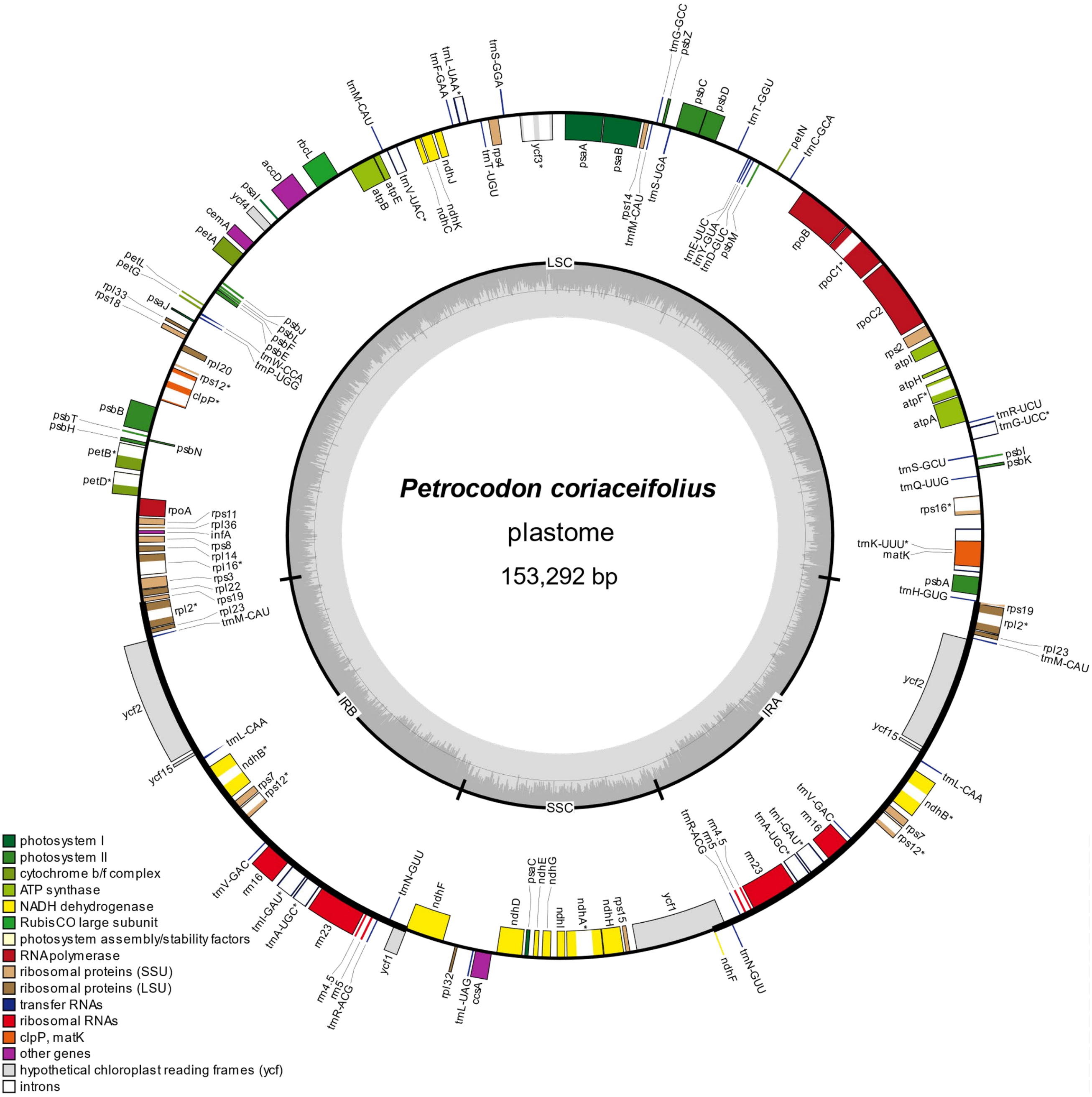

Supplementary Figure S1. The plastome map of *Petrocodon coriaceifolius*. Genes drawn on the inner side of the outer circle are transcribed clockwise, and those on the outer side are transcribed counterclockwise. IRs are shown in bold line in the outer circle. The inner circle indicates GC contents across the genome with lighter gray indicating AT contents. Genes belonging to different functional groups are shown in different colors. Gene name ends with asterisk (*) indicates the intron containing gene.

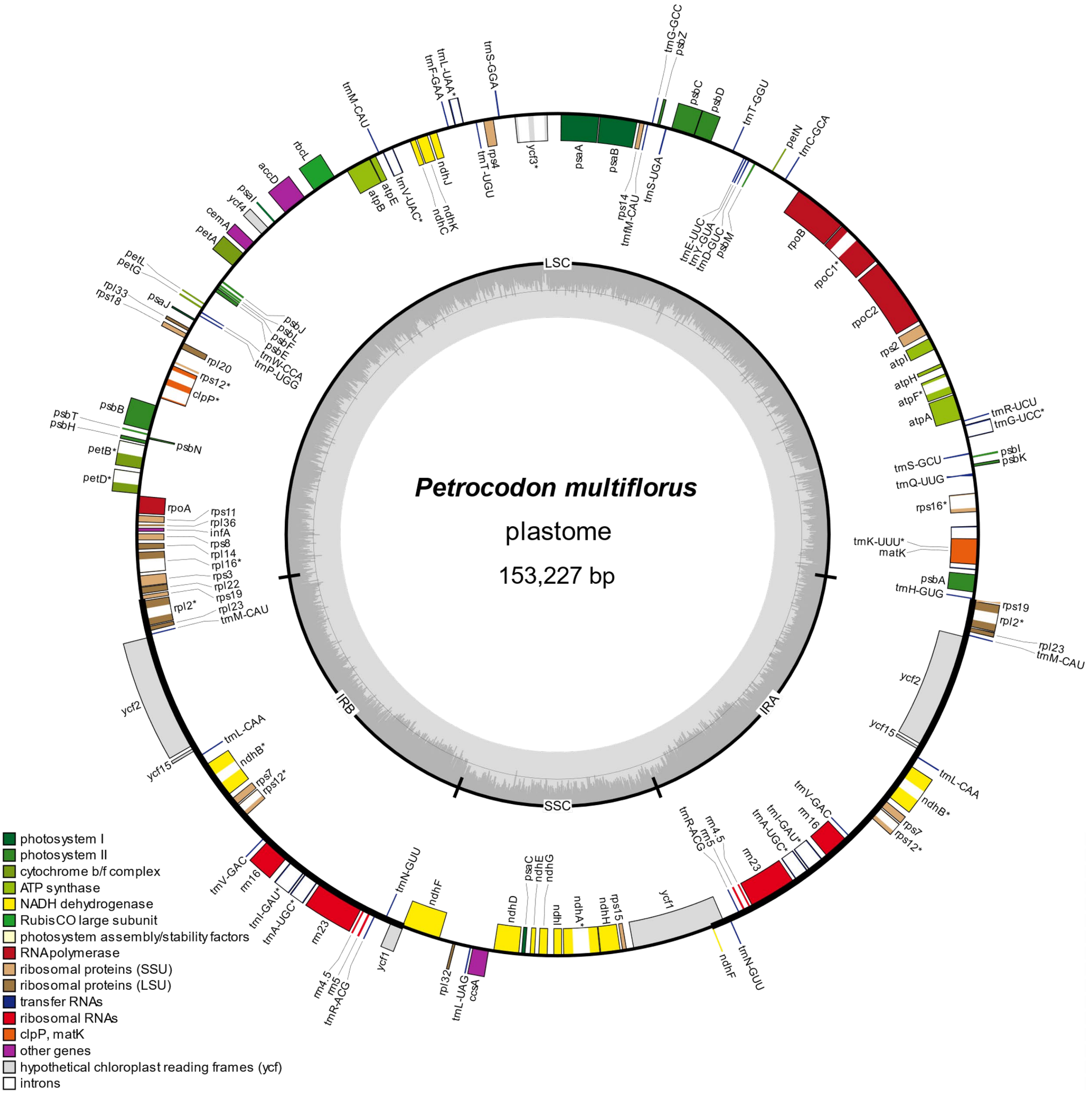

Supplementary Figure S2. The plastome map of *Petrocodon multiflorus*. Genes drawn on the inner side of the outer circle are transcribed clockwise, and those on the outer side are transcribed counterclockwise. IRs are shown in bold line in the outer circle. The inner circle indicates GC contents across the genome with lighter gray indicating AT contents. Genes belonging to different functional groups are shown in different colors. Gene name ends with asterisk (*) indicates the intron containing gene.

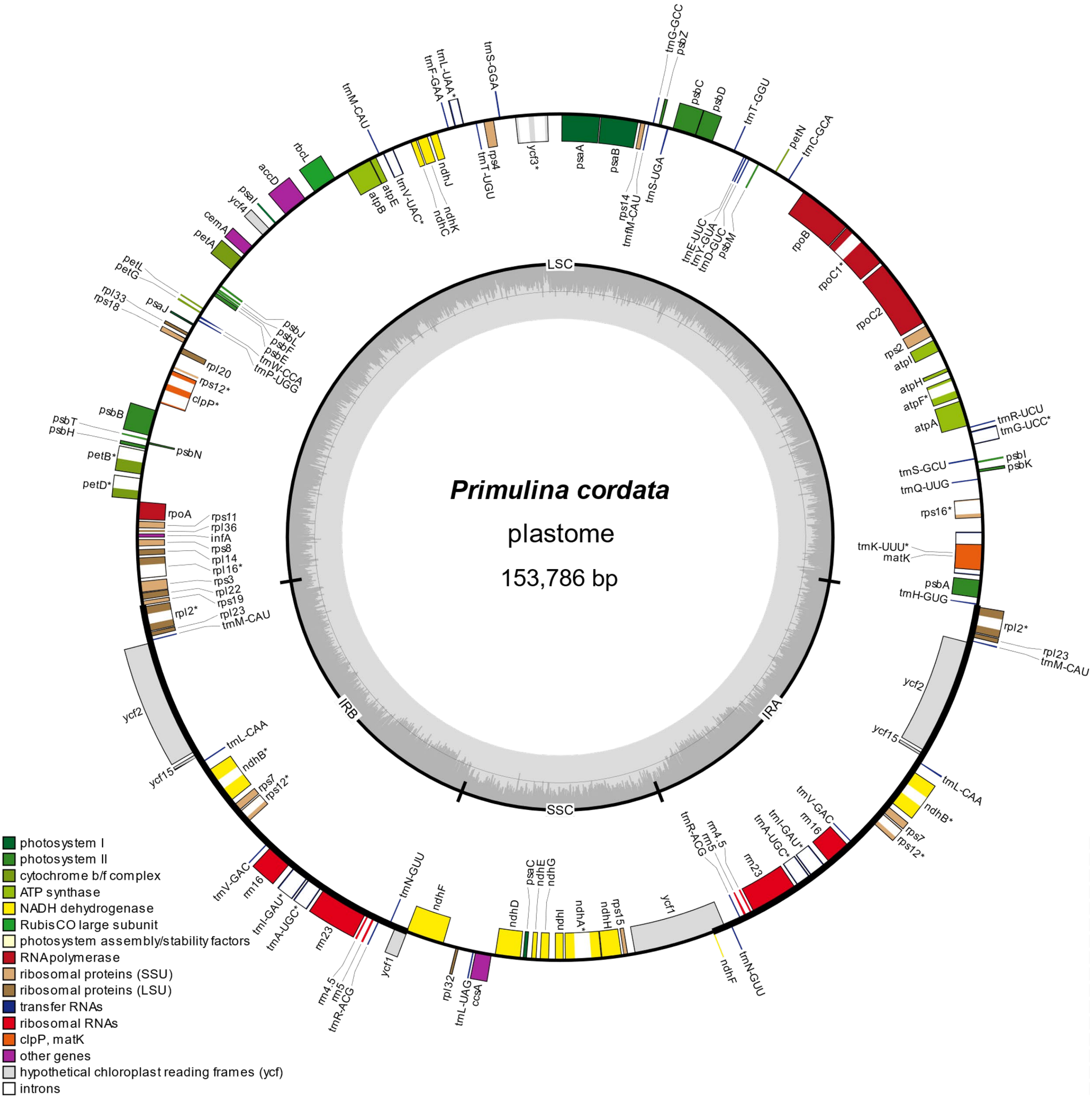

Supplementary Figure S3. The plastome map of *Primulina cordata*. Genes drawn on the inner side of the outer circle are transcribed clockwise, and those on the outer side are transcribed counterclockwise. IRs are shown in bold line in the outer circle. The inner circle indicates GC contents across the genome with lighter gray indicating AT contents. Genes belonging to different functional groups are shown in different colors. Gene name ends with asterisk (*) indicates the intron containing gene.

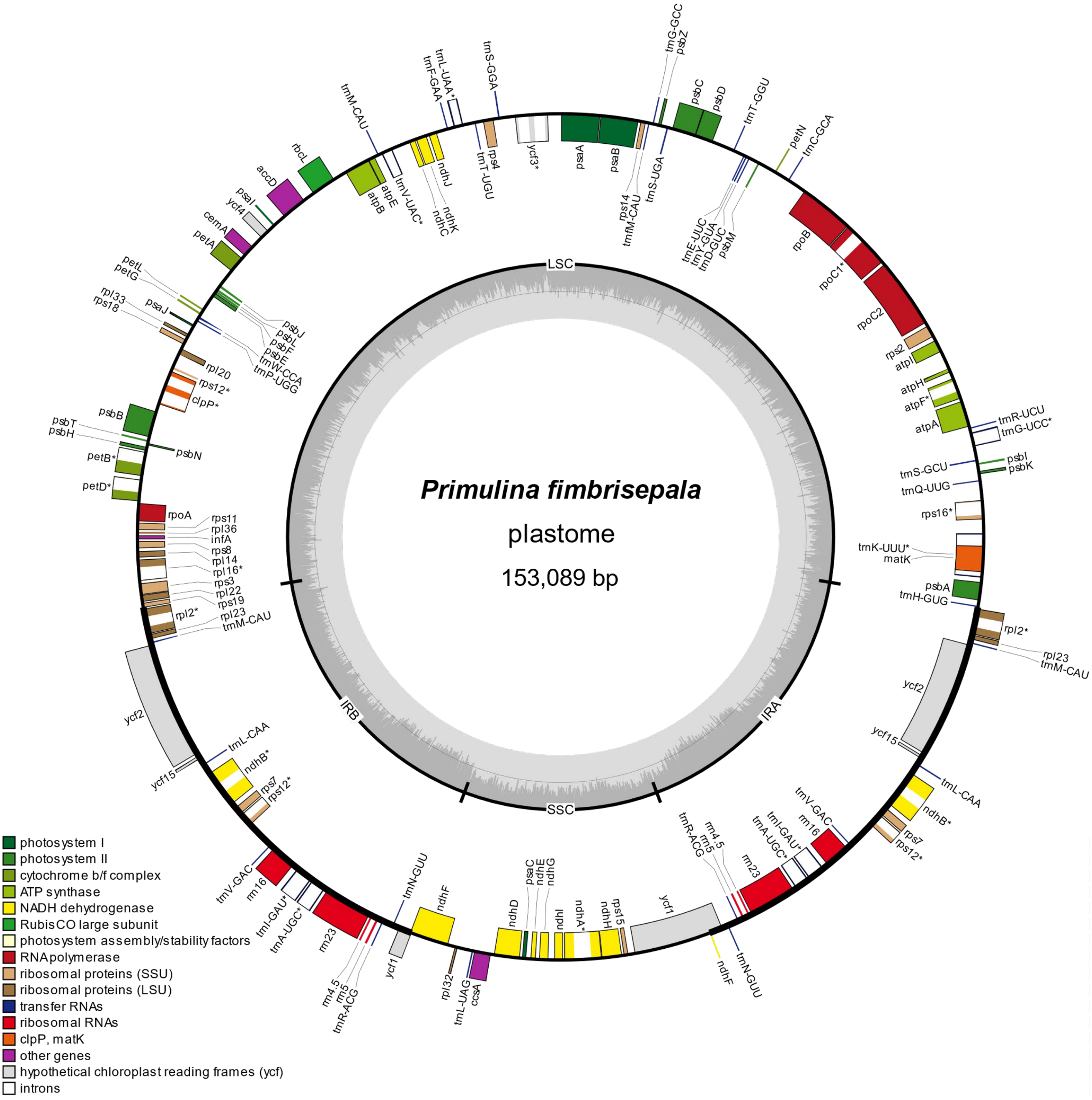

Supplementary Figure S4. The plastome map of *Primulina fimbrisepala*. Genes drawn on the inner side of the outer circle are transcribed clockwise, and those on the outer side are transcribed counterclockwise. IRs are shown in bold line in the outer circle. The inner circle indicates GC contents across the genome with lighter gray indicating AT contents. Genes belonging to different functional groups are shown in different colors. Gene name ends with asterisk (*) indicates the intron containing gene.

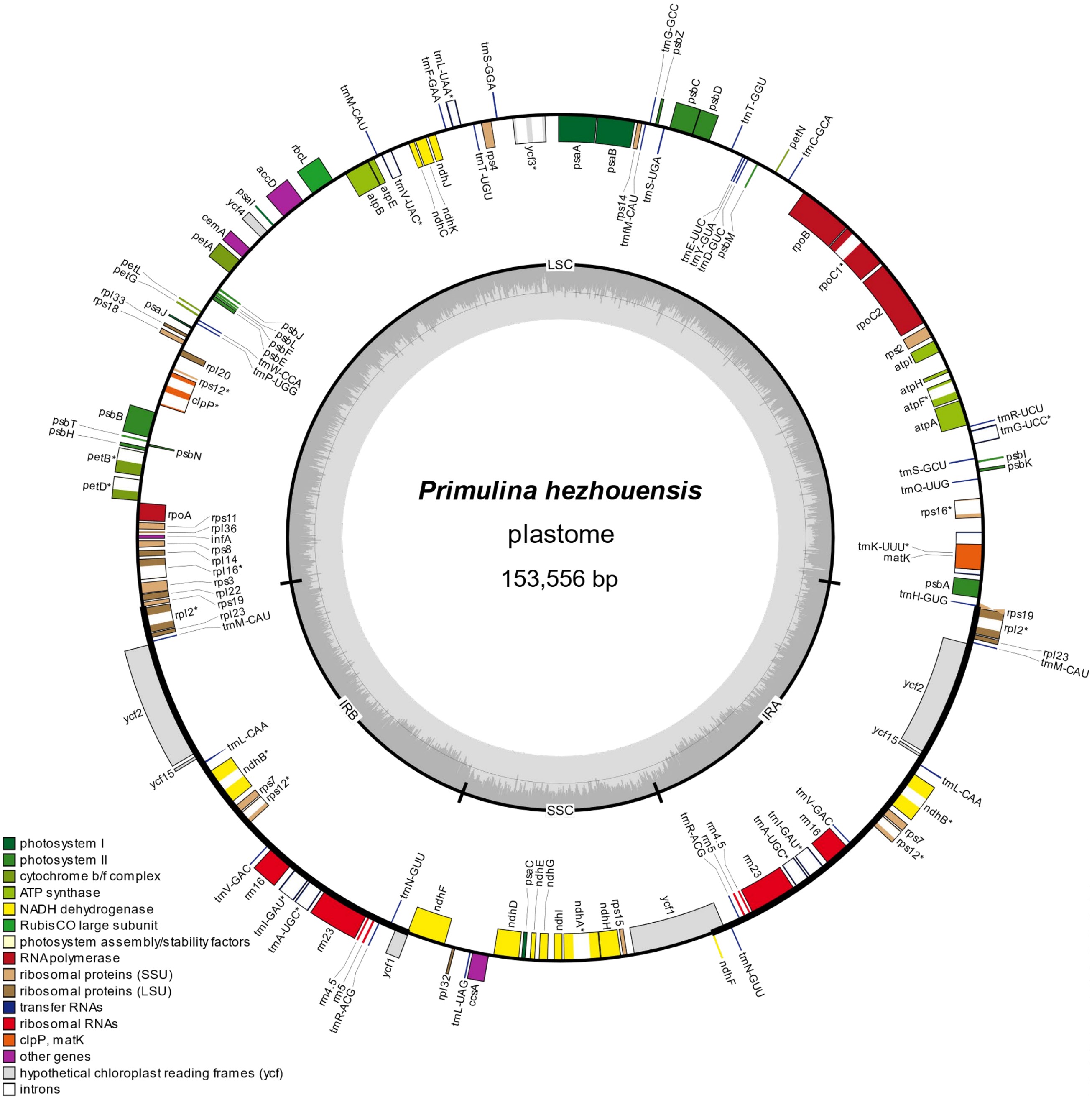

Supplementary Figure S5. The plastome map of *Primulina hezhouensis*. Genes drawn on the inner side of the outer circle are transcribed clockwise, and those on the outer side are transcribed counterclockwise. IRs are shown in bold line in the outer circle. The inner circle indicates GC contents across the genome with lighter gray indicating AT contents. Genes belonging to different functional groups are shown in different colors. Gene name ends with asterisk (*) indicates the intron containing gene.

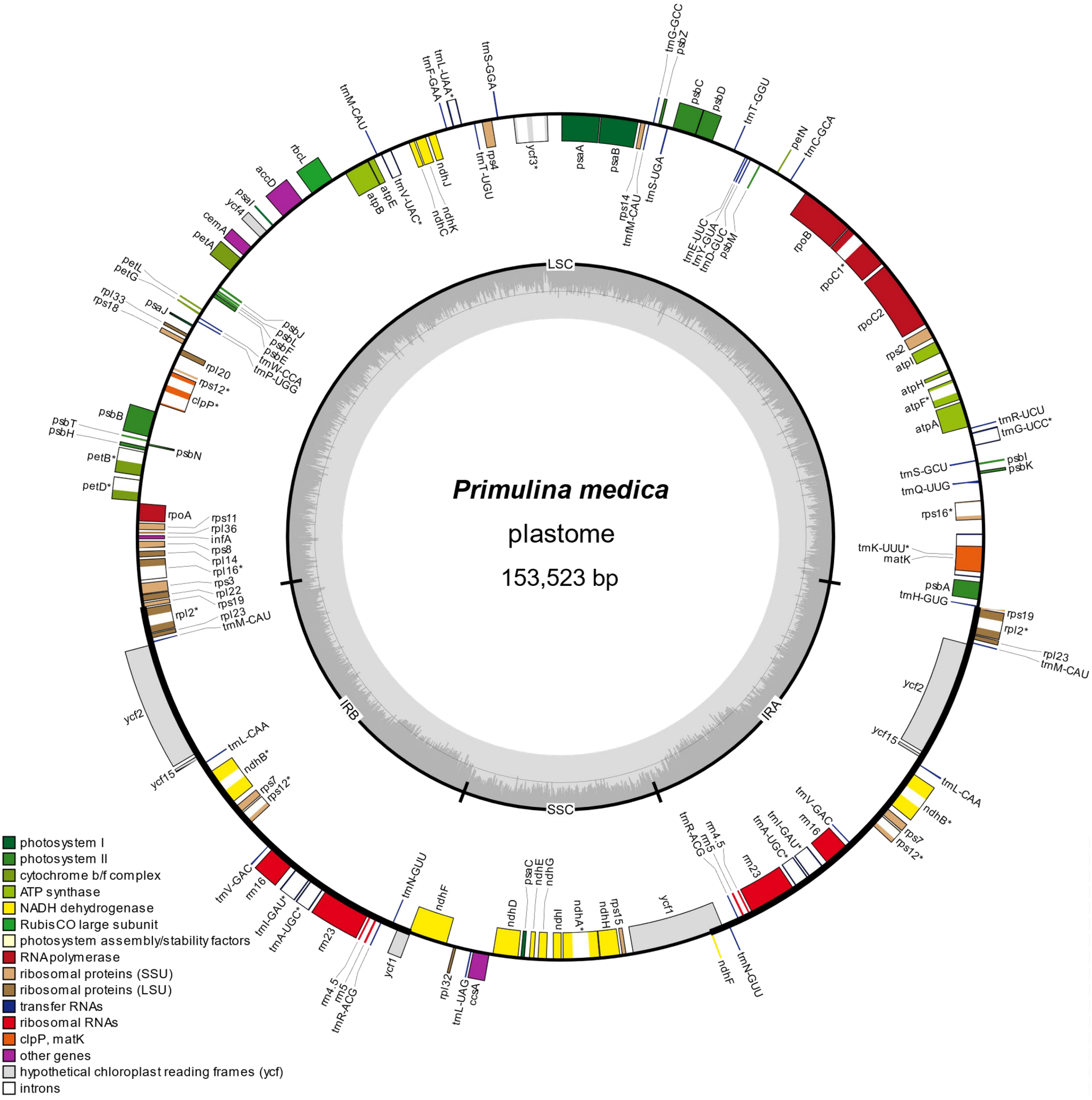

Supplementary Figure S6. The plastome map of *Primulina medica*. Genes drawn on the inner side of the outer circle are transcribed clockwise, and those on the outer side are transcribed counterclockwise. IRs are shown in bold line in the outer circle. The inner circle indicates GC contents across the genome with lighter gray indicating AT contents. Genes belonging to different functional groups are shown in different colors. Gene name ends with asterisk (*) indicates the intron containing gene.

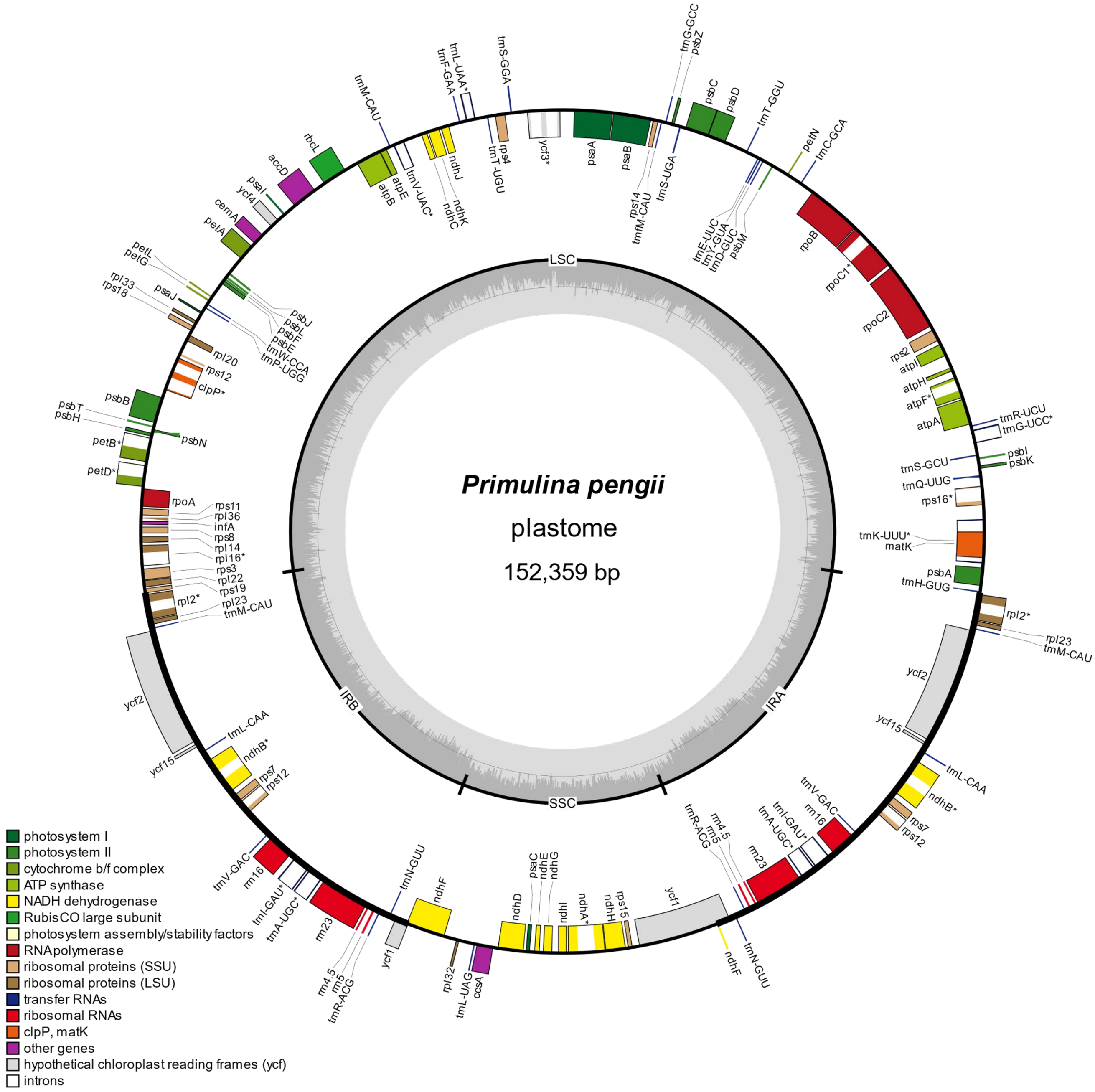

Supplementary Figure S7. The plastome map of *Primulina pengii*. Genes drawn on the inner side of the outer circle are transcribed clockwise, and those on the outer side are transcribed counterclockwise. IRs are shown in bold line in the outer circle. The inner circle indicates GC contents across the genome with lighter gray indicating AT contents. Genes belonging to different functional groups are shown in different colors. Gene name ends with asterisk (*) indicates the intron containing gene.

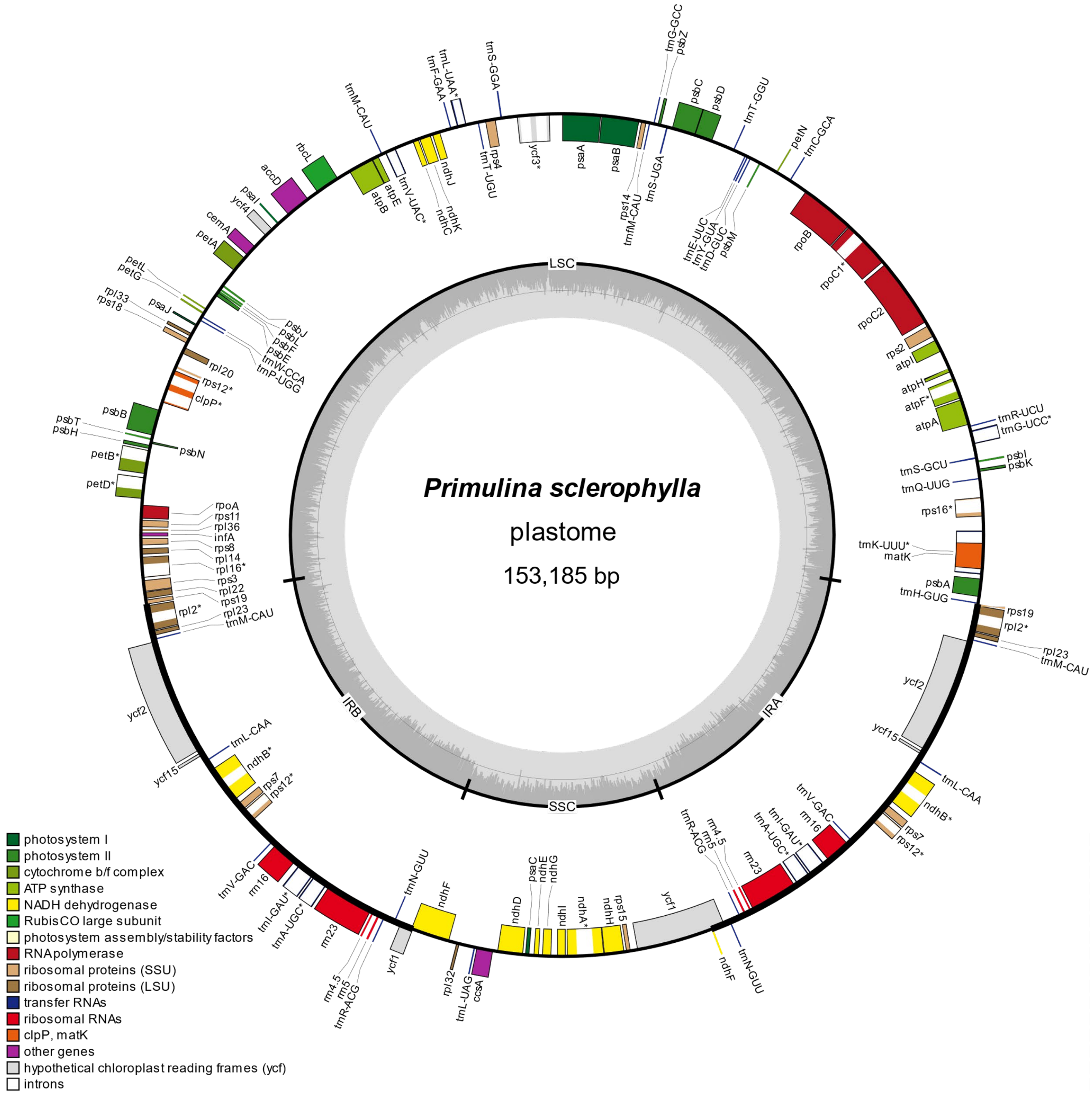

Supplementary Figure S8. The plastome map of *Primulina sclerophylla*. Genes drawn on the inner side of the outer circle are transcribed clockwise, and those on the outer side are transcribed counterclockwise. IRs are shown in bold line in the outer circle. The inner circle indicates GC contents across the genome with lighter gray indicating AT contents. Genes belonging to different functional groups are shown in different colors. Gene name ends with asterisk (*) indicates the intron containing gene.

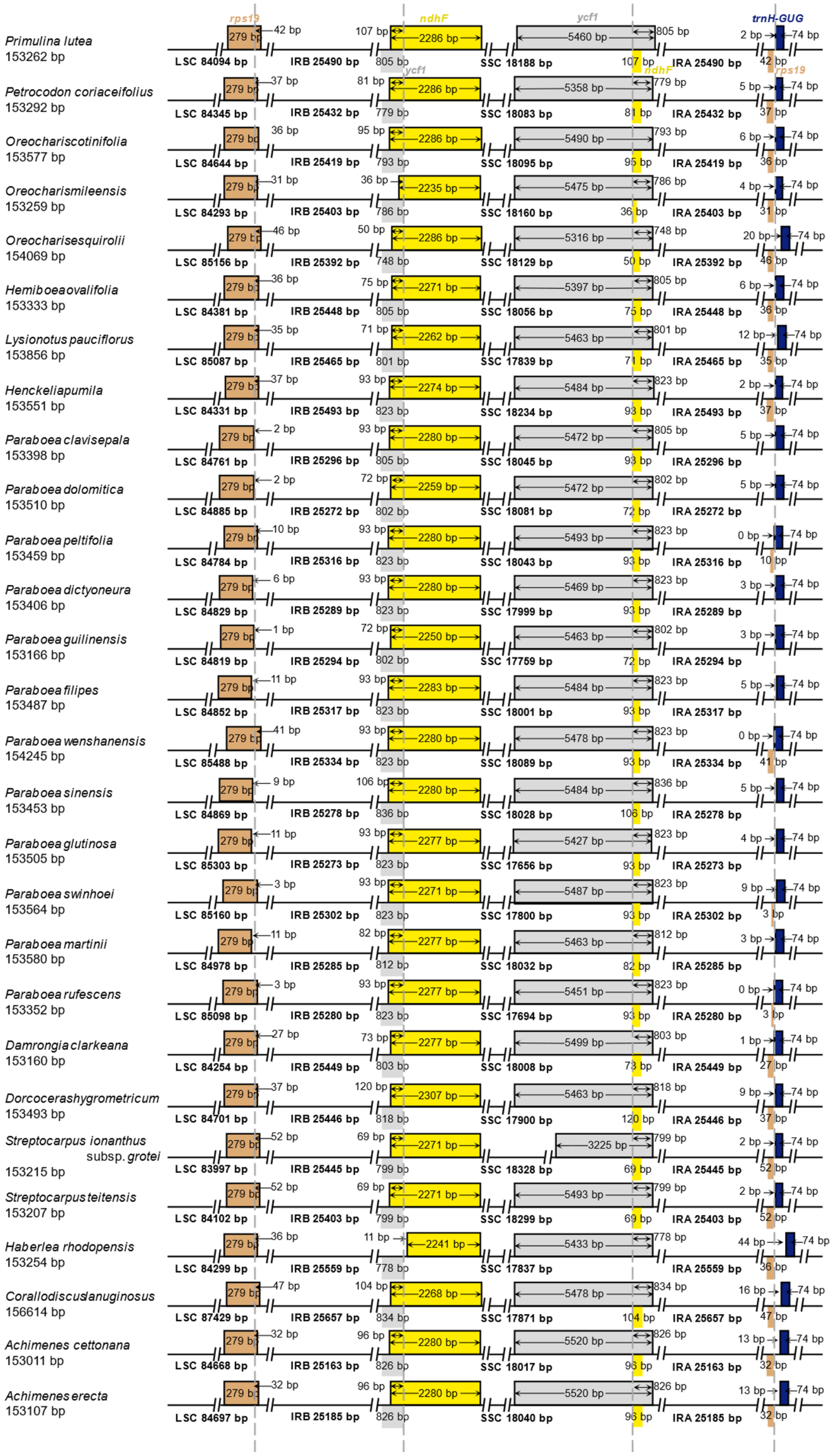

Supplementary Figure S9. The IR boundary configurations across 28 Gesneriaceae plastomes. The lengths of the boxes and lines are not in proportion to the real lengths of genes and intergenic spacers. Those gene boxes without black outline are results of partially duplication by IRs.

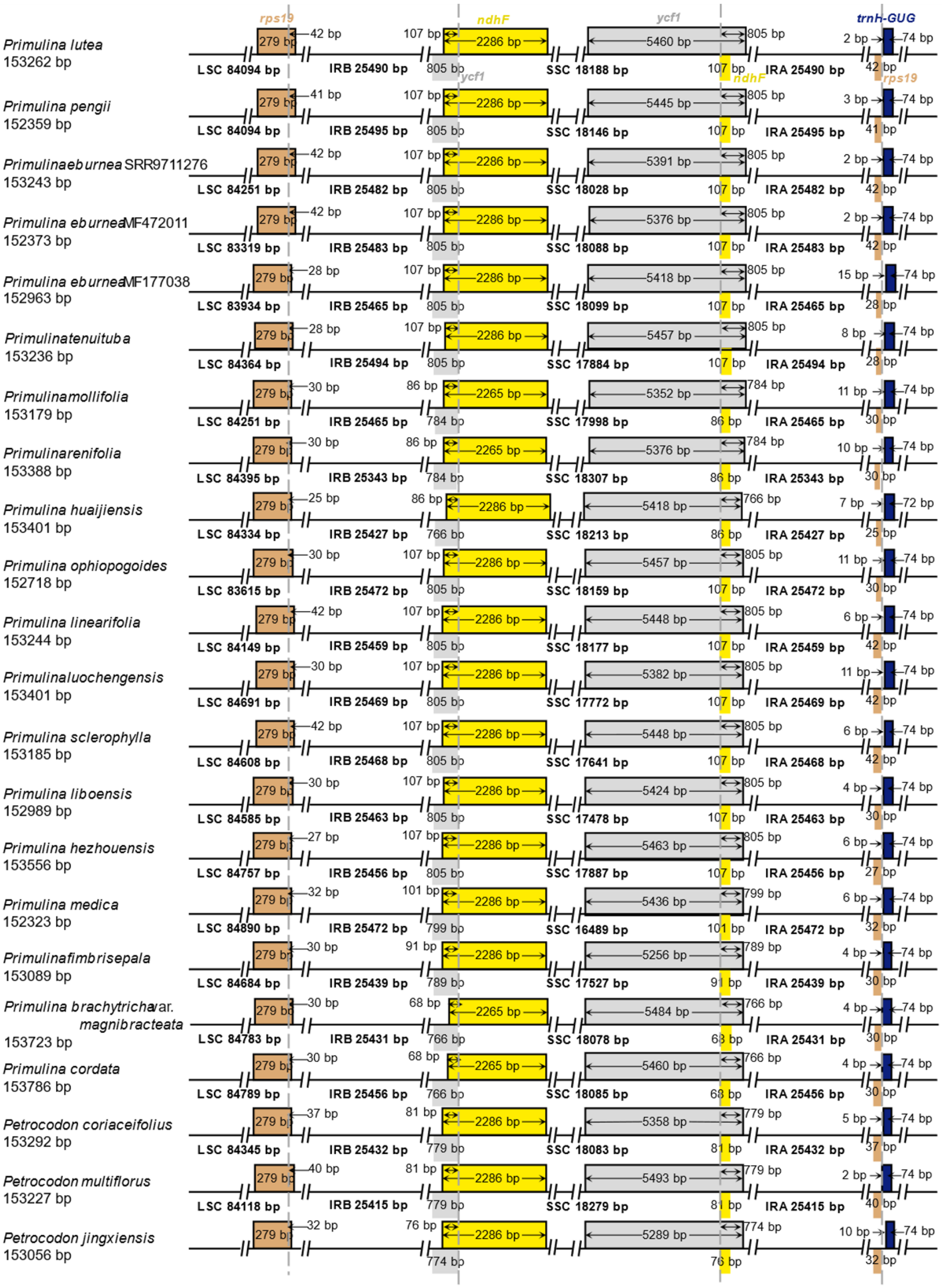

Supplementary Figure S10. The IR boundary configurations across 19 *Primulina* and 3 *Petrocodon* plastomes. The lengths of the boxes and lines are not in proportion to the real lengths of genes and intergenic spacers. Those gene boxes without black outline are results of partially duplication by IRs.

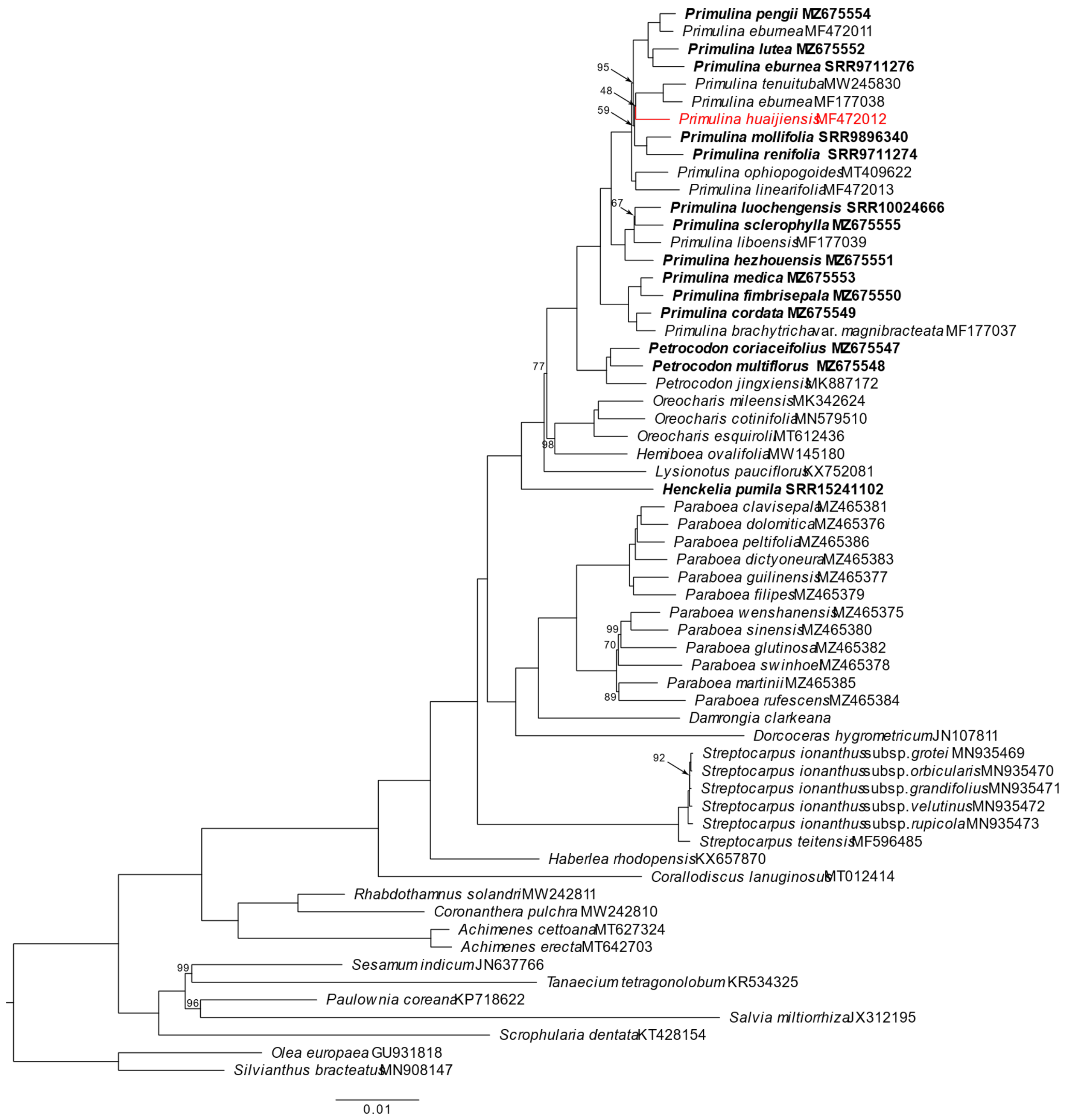

Supplementary Figure S11. The ML phylogram of 54 Gesneriaceae plastomes and seven Lamiales outgroups reconstructed by RAxML. Nodes are labeled with bootstrap support values that are not equal to 100, while those without any number labeled indicate full support value. Species name in bold indicates that the sequence is assembled by this study.

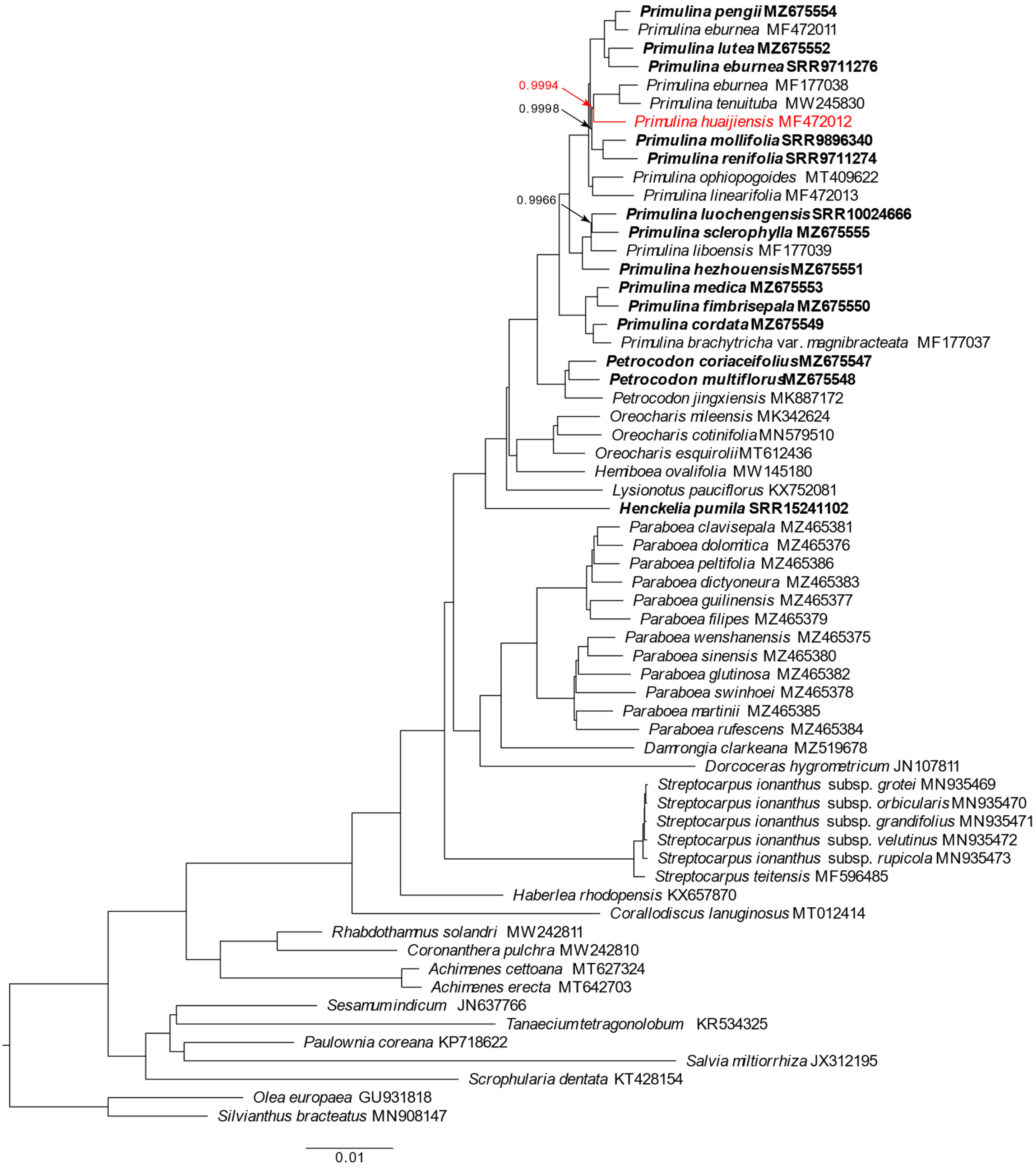

Supplementary Figure S12. The BI phylogram of 54 Gesneriaceae plastomes and seven Lamiales ourgroups reconstructed by MrBayes. Nodes are labeled with posterior probabilities that are not equal to 1.0000, while those without any number labeled indicate full support value. Species name in bold indicates that the sequence is assembled by this study.

Supplementary Tables

Supplementary Table S1. The statistics and basic information of newly assembled and published plastomes sampled in this study. (excel file)

Supplementary Table S2. SSR contents of 52 Gesneriaceae plastomes. (excel file)

Supplementary Table S3. Long repeats of 52 Gesneriaceae plastomes detected by REPuter. (excel file)

Supplementary Table S4. Observed codon usage frequency and RSCU of 52 Gesneriaceae plastomes. (excel file)

**IQ-TREE**			**MrBayes**		
**Best scheme**	**Model**	**BIC score**	**Best scheme**	**Model**	**BIC score**
CDS1	GTR+F+R3	167811.8286	CDS1	GTR+F+I+G4	168218.7711
CDS2	GTR+F+R3	146059.5523	CDS2	GTR+F+I+G4	146375.1608
CDS3	TVM+F+R4	243686.7577	CDS3	GTR+F+I+G4	244415.8228
intron	GTR+F+R4	146047.6935	intron	GTR+F+I+G4	146646.6200
RNA	TN+F+R2	26903.35107	RNA	HKY+F+I+G4	26953.69534
spacer	TVM+F+R5	530914.3210	spacer	GTR+F+I+G4	533318.6394

Supplementary Table S5. The nucleotide substitution models and the best-fit partition schemes evaluated by ModelFinder based on the Bayesian information criterion (BIC) for IQ-TREE and MrBayes analyses.
